# Near telomere-to-telomere genome assembly of Mongolian cattle: implications for population genetic variation and beef quality

**DOI:** 10.1093/gigascience/giae099

**Published:** 2024-12-18

**Authors:** Rina Su, Hao Zhou, Wenhao Yang, Sorgog Moqir, Xiji Ritu, Lei Liu, Ying Shi, Ai Dong, Menghe Bayier, Yibu Letu, Xin Manxi, Hasi Chulu, Narenhua Nasenochir, He Meng, Muren Herrid

**Affiliations:** Grassland & Cattle Investment Co., Ltd., R&D Center, Hohhot 010000, Inner Mongolia; School of Agriculture and Biology, Shanghai Jiao Tong University, Shanghai 200240, China; School of Agriculture and Biology, Shanghai Jiao Tong University, Shanghai 200240, China; Grassland & Cattle Investment Co., Ltd., R&D Center, Hohhot 010000, Inner Mongolia; Grassland & Cattle Investment Co., Ltd., R&D Center, Hohhot 010000, Inner Mongolia; Grassland & Cattle Investment Co., Ltd., R&D Center, Hohhot 010000, Inner Mongolia; Grassland & Cattle Investment Co., Ltd., R&D Center, Hohhot 010000, Inner Mongolia; Bureau of Agriculture and Animal Husbandry, Alxa League, Bayanhot 750306, Inner Mongolia, China; Centre for Animal Husbandry and Veterinary Technology, Alxa League, Bayanhot 750306, Inner Mongolia; Station for Animal Husbandry, Xilingol League, Xilinhot 026000, Inner Mongolia; Station for Animal Husbandry, Xilingol League, Xilinhot 026000, Inner Mongolia; Station for Animal Husbandry, Sunit Left Banner, Xilingol League, Xilinhot 026000, Inner Mongolia; College of Animal Science, Inner Mongolia Agriculture University, Hohhot 010000, Inner Mongolia, China; School of Agriculture and Biology, Shanghai Jiao Tong University, Shanghai 200240, China; Grassland & Cattle Investment Co., Ltd., R&D Center, Hohhot 010000, Inner Mongolia; International Livestock Research Centre, Gold Coast 4211, Queensland, Australia

**Keywords:** Mongolian cattle, near telomere-to-telomere genome, beef quality, population genetic variation

## Abstract

**Background:**

Mongolian cattle, a unique breed indigenous to China, represent valuable genetic resources and serve as important sources of meat and milk. However, there is a lack of high-quality genomes in cattle, which limits biological research and breeding improvement.

**Findings:**

In this study, we conducted whole-genome sequencing on a Mongolian bull. This effort yielded a 3.1 Gb Mongolian cattle genome sequence, with a BUSCO integrity assessment of 95.9%. The assembly achieved both contig N50 and scaffold N50 values of 110.9 Mb, with only 3 gaps identified across the entire genome. Additionally, we successfully assembled the Y chromosome among the 31 chromosomes. Notably, 3 chromosomes were identified as having telomeres at both ends. The annotation data include 54.31% repetitive sequences and 29,794 coding genes. Furthermore, a population genetic variation analysis was conducted on 332 individuals from 56 breeds, through which we identified variant loci and potentially discovered genes associated with the formation of marbling patterns in beef, predominantly located on chromosome 12.

**Conclusions:**

This study produced a genome with high continuity, completeness, and accuracy, marking the first assembly and annotation of a near telomere-to-telomere genome in cattle. Based on this, we generated a variant database comprising 332 individuals. The assembly of the genome and the analysis of population variants provide significant insights into cattle evolution and enhance our understanding of breeding selection.

## Background

Mongolian cattle originated in the Mongolian Plateau and are now distributed in regions such as Inner Mongolia, Heilongjiang, and Hebei [1]. They are an excellent breed of cattle (*Bos taurus*) and a valuable genetic resource in China [2]. In the Inner Mongolia region, which features high altitudes and dry weather, Mongolian cattle have developed strong resistance to cold, drought, and adverse conditions through long-term adaptive selection [3]. Despite the significant role of Mongolian cattle in China’s cattle industry, genomic information about this breed is relatively scarce.

With the advancement of third-generation single-molecule sequencing technology, genomic research has progressed rapidly. Currently, organisms such as humans and rice have successively achieved a telomere-to-telomere (T2T) level reference genome assembly [4, 5]. The updated genome versions offer not only more comprehensive information on genome sequences and variation maps but also valuable insights into previously challenging “genomic desert” areas, including telomeres, centromeres, and repeat sequence regions. These regions have been identified as crucial for the development of species-specific diseases and the formation of phenotypes [6].

As important economic animals, cattle contribute significantly to agriculture worldwide. Their genetic enhancement is directly correlated with advancements in genomic research. Nevertheless, current bovine reference genomes, primarily derived from European cattle breeds, suffer from several limitations, such as incomplete assemblies and identified gaps [7–9]. These deficiencies underscore the imperative for a more comprehensive and precise bovine genome assembly to inform biological studies and breeding programs, particularly for non-European cattle breeds. Currently, there is limited genomic assembly information available for Chinese indigenous cattle, leading to an incomplete understanding of their genomic characteristics. Additionally, the genetic mechanisms of cattle traits, such as meat quality, also need to be studied.

Therefore, to address the lack in the genomic research of indigenous Chinese cattle, we utilized a combination of next-generation and third-generation sequencing technologies to assemble a near T2T genome of the Mongolian cattle. Furthermore, utilizing the Mongolian assembly as a reference, we additionally resequenced 95 individuals and downloaded data for 237 individuals from public databases for population variation analysis. By comparing 56 breeds, we identified 106 million single-nucleotide polymorphisms (SNPs), 4.89 million insertions, and 5.40 million deletions, which provided us with a comprehensive understanding of the cattle population genome. It is interesting to note that, upon comparing the genomes of other cattle and Wagyu cattle, potential genes associated with the formation of beef marbling patterns were identified. The genome of indigenous Chinese cattle provides valuable insights into the genetic basis and population structure of native cattle breeds. This knowledge not only enhances the selection and breeding practices of breeders but also plays a pivotal role in the conservation efforts of local Chinese breeds.

## Results

### Construction of the high-quality sequence map

**Figure 1:**
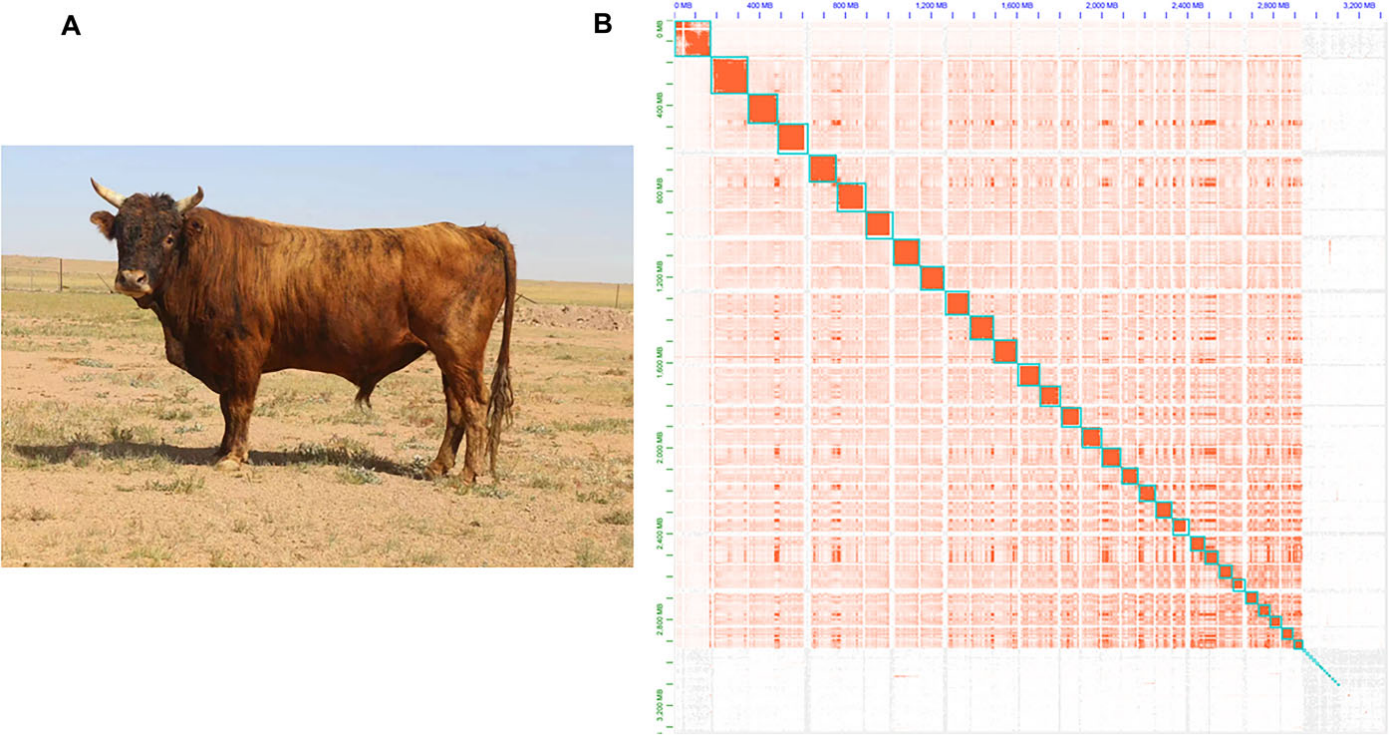
(A) Morphological photograph of Mongolian cattle. (B) Hi-C chromatin interaction map of the Mongolian cattle assembly, with chromosomes presented from top to bottom and from left to right, representing Chr1 to Chr29, ChrX.

**Figure 2:**
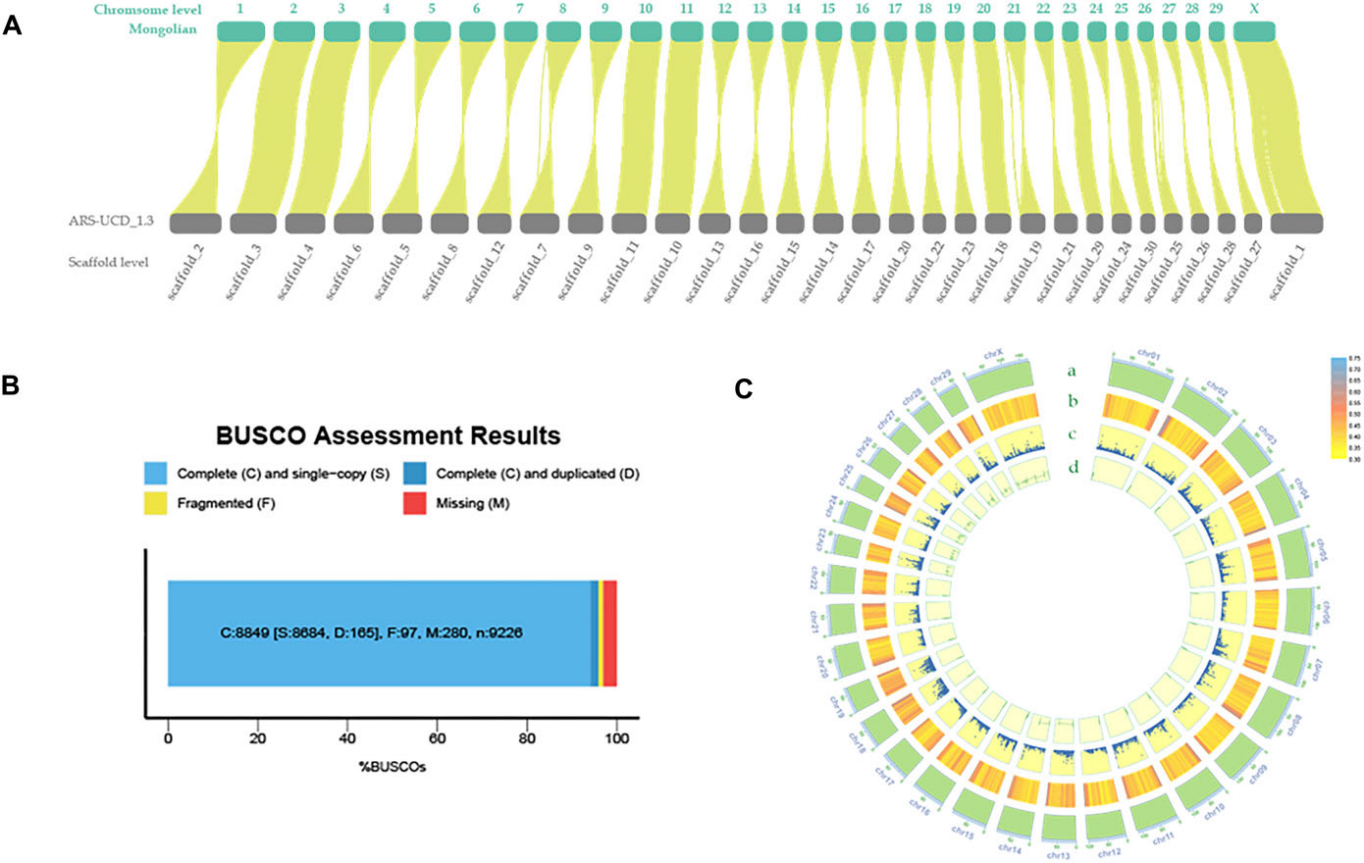
(A) The pairwise genome alignments of the Mongolian genome and the Hereford cattle genome are displayed. (B) Bar chart illustrating the BUSCO assessment of the Mongolian cattle genome. (C) The circos plot of the Mongolian cattle genome assembly. The rings from outside to inside indicate (a) chromosomes of the Mongolian genome, (b) GC density, (c) gene density, and (d) repeat density; b–d were drawn in 100-kb sliding windows.

We collected a total of 114 Gb HiFi data, 155 Gb ONT data (N50 >75 kb), and 403.6 Gb Hi-C data (Supplementary Table S1). The assembly was conducted using Hifiasm [10]. After joint assembly and redundancy removal, we obtained the final contig assembly (Supplementary Table S2). This version comprises 56 contigs with a total length of 3.1 Gb and a contig N50 of 110.9 Mb. Following auxiliary assembly using Hi-C data, 53 sequences were generated. Visualization based on scaffold interaction intensity and position revealed distinct groupings on the Hi-C heatmap (Fig. 1B). Within each grouping, interaction strength at the diagonal exceeded that at nondiagonal positions, indicating effective genome anchoring through Hi-C assistance. Notably, ptg000040l, ptg000060l, and ptg000025l assembled into the X chromosome, while ptg000039l and ptg000030l formed a chromosome. Further analysis confirmed 28 contigs fully matching cattle reference genome chromosomes (Fig. 2A). The remaining 23 contigs were inferred to belong to the Y chromosome. By aligning and assembling these contigs with the scaffold of the male cattle Y chromosome (CP128563.1), 3 contigs were assembled into the Mongolian cattle Y chromosome, resulting in a Y chromosome length of 17.9 Mb. At this point, 5 gaps remained in the genome. Gap filling addressed 1 gap in chromosome 6 and 1 gap in chromosome X, leaving 3 gaps unresolved. The final genome version after gap filling was defined as the definitive version used for subsequent analyses (Supplementary Table S3).

The contig N50 of the Mongolian cattle genome significantly surpassed the published Hereford cattle genome (Table 1). Assessment using BUSCO software indicated the assembled Mongolian cattle genome’s completeness at 95.9% (Fig. 2B), underscoring its high quality. We also assessed accuracy and completeness using Merqury [11], which yielded an assembly consensus quality value (QV) value of 54.732 and a completeness of 95.223% (Supplementary Table S4, Supplementary Fig. S1).

**Table 1:** Genome assembly statistics

Genomic features	ARS-UCD1.3	Mongolia v1.3
Total length (Gp)	2.7 Gb	3.1 Gb
Number of contigs	2,342	56
Number of scaffolds	1,956	53
Contig N50 (bp)	25.9	110.9
Scaffold N50 (Mb)	103.3	110.9
Longest contig (bp)	119,708,465	171,934,863
Longest scaffold (bp)	158,534,110	174,471,981
GC content	41.5	43.41
Number of chromosomes	30	31

Due to its complex structure, the Y chromosome has consistently posed challenges for sequencing and assembly. We conducted a synteny analysis of the assembled Y chromosome with other assembled versions, revealing high consistency and homology (Supplementary Fig. S2). Upon sequence identification of assembled scaffolds, telomeres were identified at one end of 23 chromosomes. Notably, telomeric sequences were observed at both ends of chromosome X, chromosome 21, and chromosome 25 (Supplementary Table S3), suggesting potential T2T-level assembly for these 3 chromosomes. We also used Centromics [12] and quarTeT [13] CentroMiner to identify centromeres by detecting high-copy tandem repeats, selecting candidate sites for the centromeres (Supplementary Fig. S3).

### Genome annotation information

Repeated sequences in the Mongolian cattle genome include dispersed repeats and tandem repeats. These sequences, classified as long terminal repeat (LTR), long interspersed nuclear element (LINE), short interspersed element (SINE), and DNA transposons, collectively account for 54.31% of the genome, consistent with patterns observed in mammals, validating the accuracy of repeat identification (Table 2). Following the masking of repetitive sequences, Liftoff was employed for annotation, revealing a total of 29,794 protein-coding genes (Fig. 2C, Supplementary File S1).

**Table 2:** Statistics of repetitive elements

Element Category	Element	Number of elements	Length occupied (bp)	Percentage of sequence
SINEs		2,117,318	317,605,131	10.23
	MIRs	403,380	58,075,101	1.87
LINEs		1,347,248	760,478,313	24.49
	LINE1	598,858	349,393,159	11.25
	LINE2	259,858	67,070,932	2.16
	L3/CR1	34,951	7,232,125	0.23
	RTE	452,385	336,604,273	10.84
LTR elements		474,297	163,941,172	5.28
	ERVL	76,222	30,060,567	0.97
	ERVL-MaLRs	122,838	40,272,757	1.30
	ERV_classI	92,748	40,285,943	1.30
	ERV_classII	165,418	49,313,737	1.59
DNA elements		293,384	58,196,559	1.87
	hAT-Charlie	165,710	30,826,106	0.99
	TcMar-Tigger	45,550	12,079,090	0.39
Unclassified		3,032	466,630	0.02
Total interspersed repeats			1,300,687,805	41.89
Small RNA	—	260,501	4,4038,646	1.42
Satellites	—	53,009	356,125,180	11.47
Simple repeats	—	570,811	24,190,888	0.78
Low complexity	—	85,741	4,213,890	0.14

This extensive catalog of protein-coding genes forms a critical basis for understanding genome functionality. Additionally, noncoding RNAs (ncRNAs) were identified, including 1,082 transfer RNAs (tRNAs), 955 small nuclear RNAs (snRNAs), 612 small nucleolar RNAs (snoRNAs), and 7,240 long noncoding RNAs (lncRNAs), underscoring their roles in gene regulation and epigenetic mechanisms. This comprehensive annotation sheds light on the intricate genomic architecture and sets the stage for future functional and comparative genomic investigations.

### Construction cattle genetic variation database

**Figure 3:**
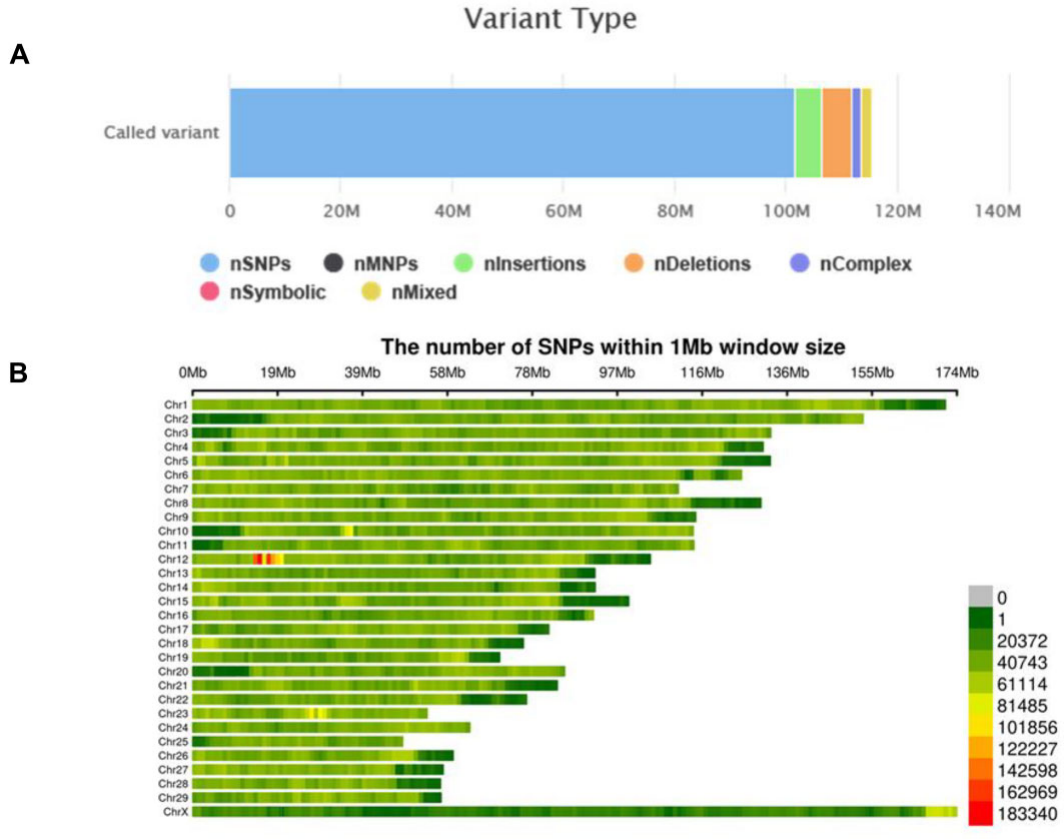
(A) Bar chart depicting the number and proportion of various variations in cattle. Among them, nSNPs account for 101,612,545 (88%), nInsertions for 4,899,369 (4.2%), nDeletions for 5,408,006 (4.7%), nComplex for 1,532,156 (1.3%), and nMixed for 1,980,238 (1.7%), with the remainder being zero. (B) Distribution of SNPs on chromosomes.

In this study, we sequenced 95 individuals from 11 cattle breeds, obtaining a total of 4.17 Tb of base data (Supplementary Table S5). Additionally, we collected data for 237 individuals from 45 breeds from the NCBI SRA database (Supplementary Table S6). Using our assembled genome as the reference, we aligned the data from 56 breeds and identified 106 million SNPs, 4.89 million insertions, and 5.4 million deletions to form a comprehensive cattle population genetic variation database (Fig. 3).

Using PopIns2 for population variant analysis, we identified a total of 674 Mb of novel nonreference sequences from 332 cattle individuals. The longest of these sequences was 1,280,529 bp, and the assembly had an N50 value of 29,590 bp. After aligning these sequences back to the cattle reference genome, we identified 2,845 structural variations (SVs). These SVs represent potentially important genetic variations that are absent from the reference genome and may contribute to breed-specific traits in cattle populations.

### Comparative genomics analysis

**Figure 4:**
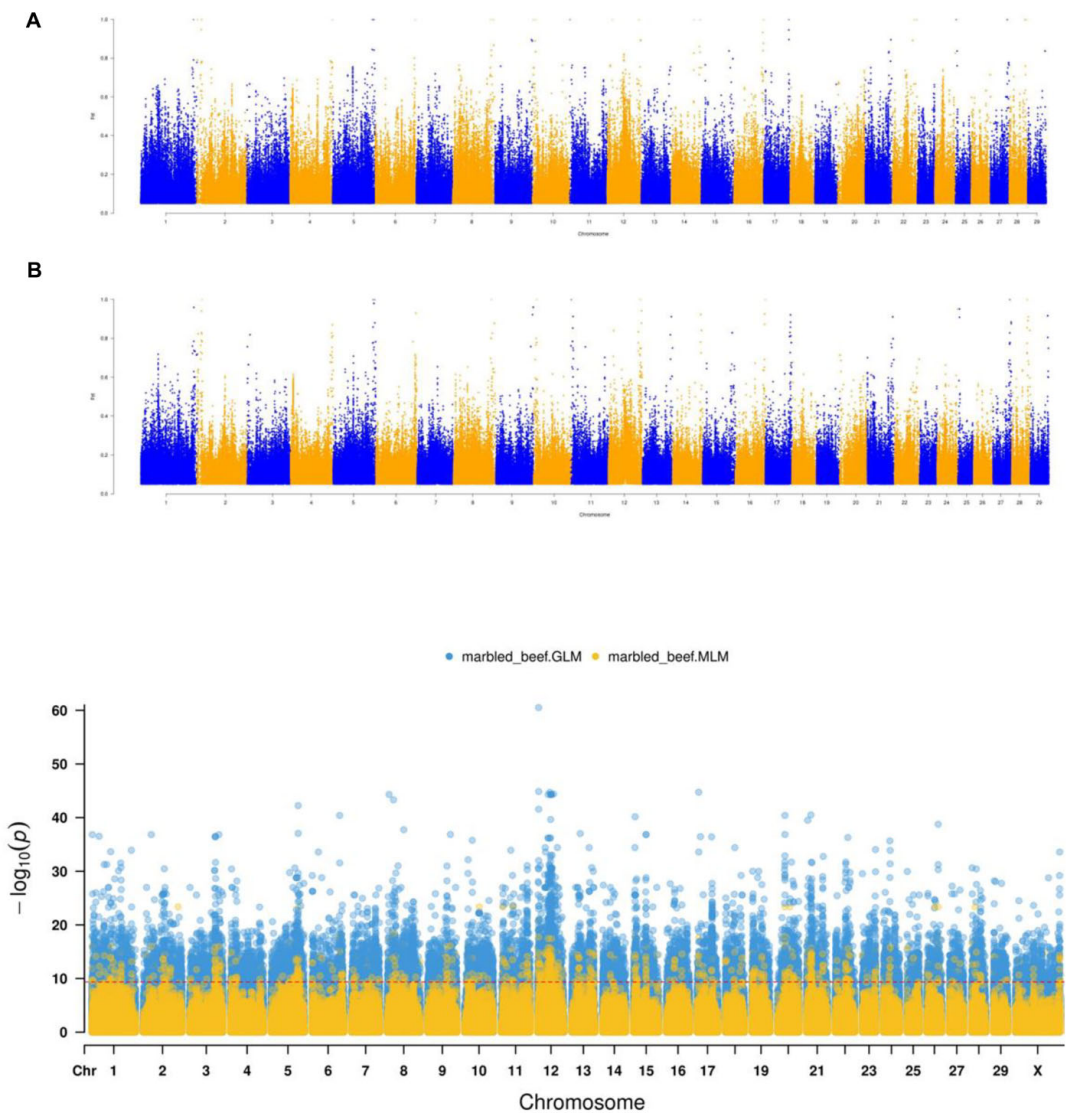
(A) Manhattan plot of the variant sites from the comparative analysis between Mongolian cattle and Wagyu. (B) Manhattan plot of the variant sites the comparative analysis between Wagyu and other beef cattle. (C) GWAS analysis of beef cattle populations using GLM and MLM models.

Despite the unique characteristics exhibited by certain cattle breeds, the underlying genetic mechanisms remain largely unknown. Wagyu cattle, renowned for their heavily marbled meat, offer superior tenderness, flavor, and juiciness, distinguishing them in the global beef market [14]. To explore genomic differences between Mongolian cattle and Wagyu, we conducted a comparative genomics analysis using the assembled Mongolian cattle genome. We identified a total of 99,429,985 common variant sites and computed Fst values (Fig. 4A). From these, 994,299 candidate sites within 735,339 genes were selected for further analysis. Functional and pathway enrichment analysis revealed 43 significantly enriched functions, including cell junction, lipid binding, long-chain fatty acid transport, and developmental growth involved in morphogenesis. Additionally, we identified 145 enriched pathways such as axon guidance, calcium signaling pathway, B-cell receptor signaling pathway, and growth hormone synthesis, secretion, and action (Supplementary Table S7).

Over years of artificial selection, Wagyu beef has developed a distinct marbling pattern. To decipher the mechanisms behind its exceptional meat quality, we conducted comparative genomics analysis between Wagyu and other beef cattle (Fig. 4B, Supplementary Table S6). Initially, we computed Fst values and discovered 95,010,853 total variants. After screening, we decided on 954,652 differential variant sites using a threshold of 0.422786. Annotation analysis revealed 18,976 candidate genes. Functional annotation of these genes found 12 significantly enriched functions such as proteolysis, lipid binding, postsynapse, and secretory granule membrane. Furthermore, 184 enriched pathways were identified, including axon guidance, focal adhesion, and cAMP signaling pathway (Supplementary Table S8).

In addition, we performed genome-wide association study (GWAS) analysis on the beef cattle population to further select relevant sites (Fig. 4C, Supplementary Table S9). This analysis identified 54,250 significant sites using both generalized linear model (GLM) and mixed linear model (MLM). Notably, a significant signal was detected on chromosome 12 of the Wagyu genome within specific intervals. This region encompasses 8 genes: *FAM155A, KLF12, KLHL1, PCDH9, ATP12A, MPHOSPH8, LOC101902228*, and *RFC3*, potentially playing crucial roles in the formation of the marbled pattern in Wagyu beef (Supplementary Table S10).

## Method

### Sample collection and sequencing

This study conducted whole-genome sequencing using blood samples from a 5-year-old Mongolian cattle in the Xilingol region. The collected samples were stored at −80°C until DNA extraction. The collection and handling of these samples were carried out in accordance with approved guidelines and regulations from Shanghai Jiao Tong University.

Library construction was carried out in accordance with the official recommendations of various sequencing platforms. The PacBio Sequel IIe (PacBio Sequel II System, RRID:SCR_017990) offers high-quality long read sequences (HiFi reads) [15]. PromethION P48 generates Ultralong Oxford Nanopore Reads (ONT reads, RRID:SCR_003756) [16]. Hi-C (chromosome conformation capture) sequencing was generates from novaseq 6000 [17]. In total, we collected 114 Gb of HiFi data, 155 Gb of ONT data (with N50 >75 kb), and 403.6 Gb of Hi-C data (Supplementary Table S1). Second-generation sequencing was obtained from the Illumina NovaSeq 6000 instrument (RRID:SCR_016387). In addition, 95 individuals generated in this study were resequenced using second-generation sequencing technology at the BGI DNBSEQ-T7 platform (RRID:SCR_017981) (Supplementary Table S5). An additional 237 individuals had their genome sequences obtained from the NCBI SRA database (Supplementary Table S6).

### Genome assembly

We utilized Hifiasm (RRID:SCR_021069) v0.16.1-r375 for genome assembly, leveraging its novel approach to construct ultralong-read overlapped graphs [10]. Initially, error-prone long reads were mapped against themselves to form an initial graph, which was then iteratively simplified by trimming tips and resolving bubbles to achieve the final assembly. This method has demonstrated efficacy in producing high-quality assemblies with substantial contig N50 values. Throughout our study, assembly parameters were meticulously adjusted to optimize genome quality based on N50 and gap count, using default settings in the software package.

Furthermore, purge_dups (RRID:SCR_021173) v1.2.5 [18] was employed to eliminate redundant heterozygous duplications, which can significantly impact assembly accuracy. This algorithm utilizes read depth information and sequence similarity to identify and remove redundant contigs, thereby improving assembly fidelity.

To scaffold the genome, we employed Chromap v0.2.5-r473 [19] and YaHS (RRID:SCR_022965) v 1.2 [20] software suites in conjunction with Hi-C data. Chromap efficiently maps high-throughput chromatin conformation capture (Hi-C) data and integrates it into the assembly process. YaHS utilizes Hi-C interaction frequencies to correct misassemblies and organize assembled sequences into clusters, ensuring accurate orientation and order. For assembling the Y chromosome, the existing cattle genome Y chromosome sequence from NCBI (CP128563.1) served as the reference scaffold, and the assembly was further refined using RagTag [21]. Quality assessment of the assembled genome was conducted using BUSCO (RRID:SCR_015008) v5.4.4 [22] (mammalia_odb10) for evaluating gene space completeness and Quast (RRID:SCR_001228) v5.1.0rc1 [23] for analyzing key genomic metrics such as GC content and total length. We used Merqury (RRID:SCR_022964) [11], a *k*-mer–based genome assessment tool, to evaluate the accuracy and completeness of this assembly. First, we created a database of the second-generation sequencing data using Meryl, followed by running Merqury for the analysis.

We downloaded 4 Y chromosome assemblies from NCBI: CM054900.2, CM037826.1, NC_082638.1, and CP128563.1. These assemblies were used to perform synteny analysis with the newly assembled Y chromosome of Mongolian cattle. For this analysis, we utilized NGenomeSyn [24], a synteny visualization tool that leverages the alignment capabilities of Minimap2 (RRID:SCR_018550) to detect and display syntenic blocks between genomes.

### Telomere and centromere identification

We used quarTeT (RRID:SCR_025258) v1.1.5 TeloExplorer to identify telomeres and CentroMiner to identify centromeres [13]. TeloExplorer detects canonical vertebrate telomere “TTAGGG” repeats across contigs, while CentroMiner identifies high-copy tandem repeats typical of centromeric regions. Due to the complex structure of centromeres, we also used Centromics to identify centromeres by detecting high-copy tandem repeats from PacBio sequencing data.

### Annotation

We employed RepeatMasker (RRID:SCR_012954) v2.0.2 [25] for detecting and masking repetitive elements within the genome, utilizing the Repbase database, the most comprehensive source of repetitive element annotations. RepeatMasker provides detailed annotations of the locations and classifications of repetitive DNAs.

Following this, Liftoff v1.6.3 [26] was utilized to transfer annotations between genomes with discrepancies, annotating protein-coding genes, lncRNAs, and small RNAs in the masked assembly. We utilized the *Bos taurus* reference genome (fa file) and its gene annotation (gff file) from the NCBI database for this purpose.

### Variant calling

The dataset included 95 individuals generated in this study, which were resequenced using second-generation sequencing technology at BGI DNBSEQ-T7, and 237 individuals whose genome sequences were obtained from public databases. The 332 clean data were aligned to our assembled reference genome using BWA-MEM 0.7.17-r1188 [27], a software that utilizes Burrows–Wheeler transform to perform rapid and precise alignment. Following this, GATK 4.3.0.0 suite [28] was employed for variant calling. The GATK pipeline includes 4 main steps: base quality score recalibration, indel realignment, duplicate removal, and variant calling. Initially, base quality scores are recalibrated to minimize machine-specific errors. Afterward, the local realignment step is done around indels to correct misalignments due to the presence of indels. Next, potential PCR duplicates are removed. Finally, the resulting cleaned, recalibrated reads are used for variant calling.

The analysis was performed using popins2 v0.13.0 [29] software suite on 332 cattle from various populations. The popins2 software provides a computational pipeline for discovering and genotyping novel sequence insertions in many individuals simultaneously. Initially, reads were aligned to the reference genome with BWA-MEM to generate BAM files. Then, a population assembly of sequences not present in the reference genome was created using FermiKit v0.13 [30]. Contigs across all individuals were merged using Minimus2 [31] into a single FASTA file. Popins2 subsequently aligned these contigs back to the reference genome and called insertion sites. Genotyping of insertion polymorphisms was performed in all individuals using a likelihood model implemented in popins2, which utilizes counts of reads supporting both the reference and insertion alleles. Finally, low-quality insertion genotypes and variants were filtered based on various quality metrics provided by the software.

### Comparative genomic analysis

Based on the obtained genetic variation dataset, comparative genomic analyses were performed. Measures of population differentiation, Fst, were computed using VCFtools 0.1.16 (RRID:SCR_001235) [32]. SnpEff v48.0 (RRID:SCR_005191) [33] was deployed for predicting the effects of identified variants while Gene Ontology and KEGG pathway analyses were conducted using the R package clusterProfiler v4.0 [34]. For deeper insights into the distinct marbled beef characteristic of Wagyu cattle, GWASs were performed using FarmCPU v1.02 [35], an R package for multiple locus mapping.

## Discussion

With the introduction of third-generation single-molecule sequencing technology, we have achieved substantial advancements in bovine genome assembly. Our assembled Mongolian cattle genome exhibits a high quality with a contig N50 of 110.9 Mb and only 3 remaining gaps. Previous studies predominantly relied on European cattle reference genomes, potentially introducing biases in the analysis of Asian cattle data [36]. Our study not only presents an improved bovine genome assembly characterized by fewer gaps compared to previous assemblies but also greatly contributes to future genetic research on Asian cattle breeds. Furthermore, our insights into telomeres and centromeres, crucial elements for chromosomal stability and cell division, add valuable knowledge to this limited research field.

Existing cattle genetic variation databases are pivotal for investigating population genetics, breed improvements, and disease resistance. Our comprehensive database, comprising data from 56 cattle breeds worldwide, contains 106 million SNPs, establishing it as the most extensive bovine genetic variation database to date. Our high-quality assembled genome helped us identify an even greater number of nonreference sequences (674 Mb), thereby providing a more precise representation of the bovine genomic diversity.

The distinctive marbling traits of Wagyu beef are highly prized, yet their genetic underpinnings remain poorly understood [14]. Through our updated genome assembly, we identified significant genetic signals harboring genes like FAM155A, KLF12, KLHL1, PCDH9, ATP12A, MPHOSPH8, LOC101902228, and RFC3. Notably, PCDH9 influences lipid metabolism, potentially impacting fat deposition and marbling in beef [37]. Similarly, ATP12A is involved in muscle pH regulation, affecting meat quality [38]. KLHL1 is associated with skeletal muscle development, possibly impacting cattle growth [39]. Exploring these genes promises valuable insights into the genetic basis of beef quality, facilitating targeted breeding and management strategies.

In conclusion, this study marks a significant advancement in bovine genomics by providing a high-quality reference genome for Mongolian cattle and shedding light on crucial genetic elements such as telomeres and centromeres. By overcoming gaps and biases associated with previous European-centric studies, our findings pave the way for more precise genetic research and breeding programs tailored to Asian cattle breeds. Furthermore, the identification of key genes associated with Wagyu beef marbling, including PCDH9, ATP12A, and KLHL1, underscores their potential roles in shaping meat quality traits. These insights not only enhance our understanding of beef production but also offer actionable knowledge for improving genetic selection strategies in cattle breeding worldwide.

## Supplementary Material

giae099_Supplemental_Files

giae099_GIGA-D-24-00289_Original_Submission

giae099_GIGA-D-24-00289_Revision_1

giae099_GIGA-D-24-00289_Revision_2

giae099_Response_to_Reviewer_Comments_Original_Submission

giae099_Response_to_Reviewer_Comments_Revision_1

giae099_Reviewer_1_Report_Original_Submission Yang Zhou -- 8/15/2024

giae099_Reviewer_1_Report_Revision_1 Yang Zhou -- 9/30/2024

giae099_Reviewer_1_Report_Revision_2 Yang Zhou -- 10/16/2024

giae099_Reviewer_2_Report_Original_Submission Yandong Ren, Ph.d -- 8/15/2024

## Data Availability

The Mongolian cattle genome assembly, as reported in this article, has been deposited in GenBank under the project PRJNA1140538 with the accession number JBFSJU000000000.1. The raw sequences used for the genome assembly have been deposited in NCBI under the project PRJNA1140538, with accession numbers SRR30013109 to SRR30013112. The resequencing data have deposited in NCBI under the project PRJNA1141206. All supporting data and materials are available in the *GigaScience* database, GigaDB [40].

## Additional Files

**Supplementary Fig. S1**. Mongolian_spectra-cn plots.fl.pdf.

**Supplementary Fig. S2**. Ragtag_YvsOther.pdf.

**Supplementary Fig. S3**. Centromics_circos.pdf.

**Supplementary Table S1**. Information of raw data.xlsx.

**Supplementary Table S2**. Assembly.xlsx.

**Supplementary Table S3**. Scaffold.xlsx.

**Supplementary Table S4**. QV-assessment.xlsx.

**Supplementary Table S5**. 95_Individuals .xls.

**Supplementary Table S6**. Information of cattle population.xlsx.

**Supplementary Table S7**. Mong_vs_Wagyu.xlsx.

**Supplementary Table S8**. Wagyu_vs_other.xlsx.

**Supplementary Table S10**. Wagyu_vs_other-gwas.xlsx.

## Abbreviations

BUSCO: Benchmarking Universal Single-Copy Orthologs; GLM: generalized linear model; GWAS: genome-wide association study; KEGG: Kyoto Encyclopedia of Genes and Genomes; LINE: long interspersed nuclear element; lncRNA: long noncoding RNA; LTR: long terminal repeat; MLM: mixed linear model; ncRNA: noncoding RNA; SINE: short interspersed element; snoRNA: small nucleolar RNA; SNP: single-nucleotide polymorphism; snRNA: small nuclear RNA; T2T: telomere-to-telomere; tRNA: transfer RNA.
