# Supplementary figures and images for: Near telomere-to-telomere genome assembly of Mongolian cattle: implications for population genetic variation and beef quality
Source: Gigascience. 2024 Dec 18;13:giae099. doi: 10.1093/gigascience/giae099 (PMC11653892; doi:10.1093/gigascience/giae099)

NC\_082638.1

CM037826.1

Monglian\_Y

CM054900.2

CP128563.1

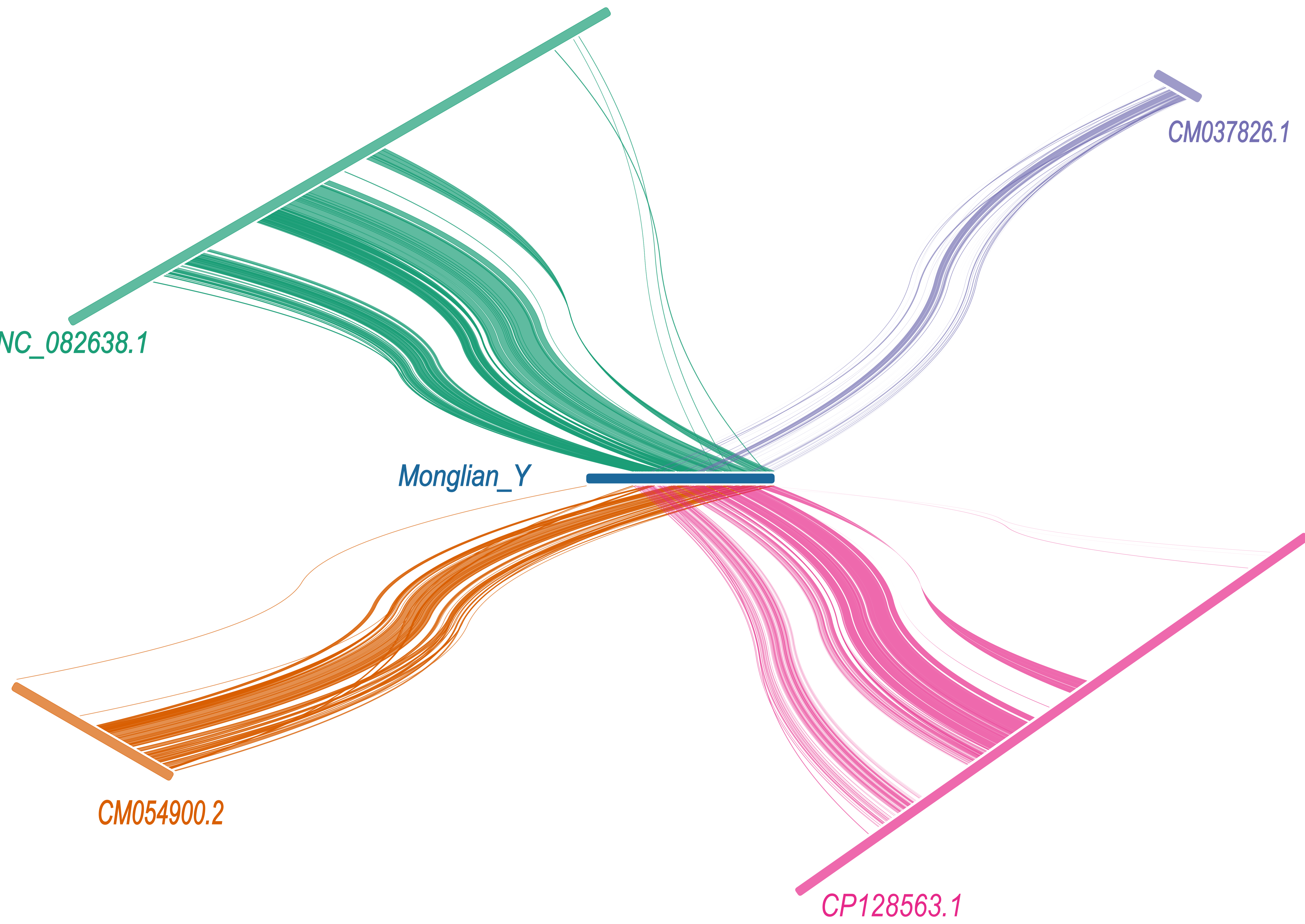

Supplement: giae099_Supplemental_Files [file giae099_supplemental_files.zip › Figure S2 Ragtag_YvsOther.pdf]

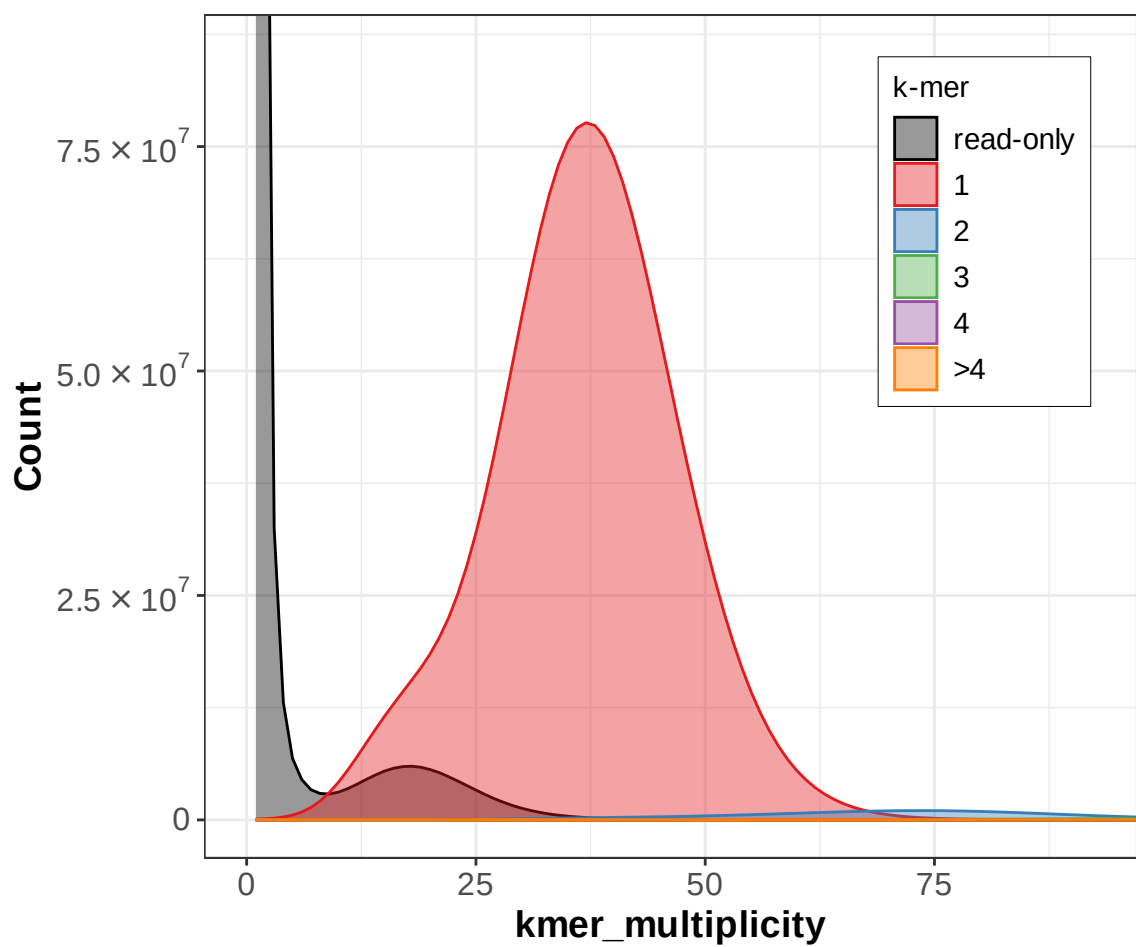

Supplement: giae099_Supplemental_Files [file giae099_supplemental_files.zip › Figure-S1.Mongolian_spectra-cn plots.fl.pdf]
