# Supplementary material for: Near telomere-to-telomere genome assembly of Mongolian cattle: implications for population genetic variation and beef quality
Source: Gigascience. 2024 Dec 18;13:giae099. doi: 10.1093/gigascience/giae099 (PMC11653892; doi:10.1093/gigascience/giae099)
Supplement: giae099_GIGA-D-24-00289_Revision_1 [file giae099_giga-d-24-00289_revision_1.pdf]

GigaScience

Near Telomere-to-Telomere Genome Assembly of Mongolian Cattle: Implications for Population Genetic Variation and Beef quality

--Manuscript Draft--

**Manuscript Number:**	GIGA-D-24-00289R1
**Full Title:**	Near Telomere-to-Telomere Genome Assembly of Mongolian Cattle: Implications for Population Genetic Variation and Beef quality
**Article Type:**	Data Note
**Funding Information:**	
**Abstract:**	Abstract<br><br>Background<br><br>Mongolian cattle, a unique breed indigenous to China, represent valuable genetic resources and serve as important sources of meat and milk. However, there is a lack of high-quality genomes in cattle, which limits biological research and breeding improvement.<br><br>Findings<br><br>In this study, we conducted whole-genome sequencing on a Mongolian bull. This effort yielded a 3.1 Gb Mongolian cattle genome sequence, with a BUSCO integrity assessment of 95.9%. The assembly achieved both Contig N50 and Scaffold N50 values of 110.9 Mb, with only 3 gaps identified across the entire genome. Additionally, We successfully assembled the Y chromosome among the 31 chromosomes. Notably, 3 chromosomes were identified as having telomeres at both ends. The annotation data includes 54.31% repetitive sequences, 29,794 coding genes. Furthermore, a population genetic variation anlysis was conducted on 332 individuals from 56 breeds, through which we identified variant loci and potentially discovered genes associated with the formation of marbling patterns in beef, predominantly located on chromosome 12.<br><br>Conclusions<br><br>This study produced a genome with high continuity, completeness, and accuracy, marking the first assembly and annotation of a near telomere-to-telomere genome in cattle. Based on this, we generated a variant database comprising 332 individuals. The assembly of the genome and the analysis of population variants provide significant insights into cattle evolution and enhance our understanding of breeding selection.
**Corresponding Author:**	He Meng<br>Shanghai Jiao Tong University School of Agriculture and Biology<br>Shanghai, Shanghai CHINA
**Corresponding Author Secondary Information:**	
**Corresponding Author's Institution:**	Shanghai Jiao Tong University School of Agriculture and Biology
**Corresponding Author's Secondary Institution:**	
**First Author:**	Wenhao Yang
**First Author Secondary Information:**	
**Order of Authors:**	Wenhao Yang
	Rina Su
	Hao Zhou
	Sorgog Moqir

Powered by Editorial Manager® and ProduXion Manager® from Aries Systems Corporation

	Xiji Ritu
	Lei Liu
	Ying Shi
	Ai Dong
	Menghe Bayier
	Yibu Letu
	Xin Manxi
	Hasi Chulu
	Narenhua Nasenochirb
	He Meng
	Muren Herrid
**Order of Authors Secondary Information:**	
**Response to Reviewers:**	Dear Editor and Reviewers:<br><br>Thank you for the opportunity to revise our manuscript, "Near Telomere-to-Telomere Genome Assembly of Mongolian Cattle: Implications for Population Genetic Variation and Beef Quality" (GIGA-D-24-00289). We appreciate the reviewers' constructive feedback and valuable suggestions, and we are committed to addressing all the points raised to improve the quality of our work.<br>We will carefully address each of the reviewers' comments in a point-by-point manner. For any revisions that we do not agree with, we will provide detailed explanations and justifications. Additionally, we will ensure that our revised manuscript complies with the journal's style guidelines.<br>Thank you again for considering our manuscript for publication in GigaScience.<br><br>Best wishes，<br>He Meng<br><br>Reviewer #1: Su et al. present a high-quality Mongolian cattle genome with long reads and Hi-C data. The genome supersedes the current reference genome ARS-UCD1.3 and other published genome at NCBI, with higher continuity and completeness.<br>Response：Thank you for your positive feedback on our work. We are pleased that our high-quality Mongolian cattle genome, assembled with long reads and Hi-C data, surpasses the current reference genome ARS-UCD1.3 and other published genomes at NCBI in terms of continuity and completeness. We hope that these data will provide strong support for future genetic research and breeding efforts.<br>1.However, according to the description in the manuscript, only three chromosomes have telomere at both ends. No QV and coverage assessment have been performed. In addition, the process of Y chromosome assembling may be problematic since Y chromosome between species and even individuals could be highly variable in structure.<br>Response：Thank you for your valuable suggestions. We have added QV assessment to the manuscript and included the relevant figures in the supplementary materials in line 126-128 . As you pointed out, the assembly of the Y chromosome is not fully refined, we have reassembled the Y chromosome to improve the accuracy and revised the description of the Y chromosome assembly in the manuscript accordingly. We have compared it with those from other cattle breeds, showing high consistency and homology in line 129- 131.<br>2.Besides, in Table S3 I notice that about 100 Mb sequence remained unanchored. Where should they belong? Please make some additional analysis.<br>Response：Thank you for pointing this out. We have analyzed the 100 Mb of unanchored sequence, and most of it consists of telomeric fragments. We did not include this information in the manuscript, as it is not the primary focus of our study and is a common phenomenon observed in genome assemblies. We appreciate your understanding on this matter.<br>3.In addition, the centromere presentation is largely missing in the manuscript. In the absence of throughout QC of the genome, I don't believe CentroMiner is a good tool to

predict centromere candidates. The tool selects the tandem repeat monomers likely to be centromeric repeats on each chromosome based on period and copy number.
Response：Thank you for your valuable suggestion. We have added a description of the centromeres in the revised manuscript. Additionally, we utilized Centromics to enhance our predictions of the centromeres, and our results are consistent with those obtained using CentroMiner. (line 136-138)
4.Therefore if the centromere are collapsed in the genome, which usually happen during genome assembling, the use of CentroMiner would result in false positive predictions. Have the authors examined the monomer sequence?
Response: Thank you for your valuable suggestion. We have examined the centromere monomer sequences and performed the analysis accordingly. The result files have been uploaded for further review.(line136-138)
5. In the analysis of the Wagyu beef marbling trait, to show the advantage of the new genome, I would suggest generating a variation dataset with the current reference ARS-UCD1.3 as well with the same set of parameters and compare the dataset with the variation dataset of the Mongolian reference genome. I doubt that the genes mentioned in the manuscripts can also be identified with ARS-UCD1.3. How many of them were newly reported in this study?
Response: Thank you for your valuable suggestion. While generating a variation dataset using the ARS-UCD1.3 reference could certainly provide useful comparative insights, our study is primarily focused on highlighting the distinctiveness and relevance of the Mongolian reference genome in relation to the Wagyu beef marbling trait. Taking into account both the scope of our research and computational considerations, we believe our current approach is the most suitable for the objectives of the study. We hope for your kind understanding.

Minor Review
1.Line132-138: liftoff only provide gene annotation transfer to the new genome, but would there be any new genes in the Mongolian genome?
Response: Thank you for your insightful suggestion. You are absolutely right that identifying novel genes in the Mongolian genome could provide additional valuable insights. However, given the completeness of current cattle genome annotations, we chose to focus on ensuring accurate gene annotation through Liftoff in this study. We believe this approach aligns best with the intended scope of our research. We appreciate your understanding.
2.Line 183: present the full name of GLM and MLM
Response: Thank you for your feedback. I have added the full names of GLM and MLM in the manuscript for clarity.
3.Line285-286, "Our study not only presents the first gapless bovine genome". There are at least 8 gaps in the genome and many scaffolds remained unanchored. Please revise.
Response: We have revised the statement to clarify that our study presents an improved bovine genome assembly characterized by fewer gaps compared to previous assemblies. We appreciate your suggestion in helping us enhance the clarity of our work.

Reviewer #2: In this manuscript, the authors provide a near telomere-to-telomere genome assembly of Mongolian cattle and a population genetic variation analysis. Most notably, the authors successfully assembled the Y chromosome and identified variant loci which may associate with the formation of marbling patterns in beef. Basically, this work is well described and the methods appropriate to the aims of the study. The description of dataset and workflows are clear and solid. However, the manuscript has some deficiencies that authors need to amend and address. Below are just the major and minor comments for this manuscript.
Due to the article type is data note, and there is no requirement of biological analyses for this manuscript. My recommendation on this manuscript is minor revision.
Response:
Thank you for your positive feedback and constructive comments regarding our manuscript. We are pleased to hear that you found our work on the near telomere-to-telomere genome assembly of Mongolian cattle and the population genetic variation analysis to be well described and methodologically appropriate.
We appreciate your recognition of the clarity and solidity of our dataset and workflow descriptions. We will carefully consider your suggestions and address the deficiencies you have pointed out in our revision.

Powered by Editorial Manager® and ProduXion Manager® from Aries Systems Corporation

	1.The Y chromosome is one of the most critical highlights of this manuscript, so the structure of Y chromosome is very important. Almost all the published papers which successful assembled the Y chromosome or just partial Y chromosome, all these papers expounded the structure Y chromosome. So, maybe the author can add this content, or at least compared the structure of Y chromosome between Mongolian cattle and other mammals.<br>Response: Thank you for your valuable suggestion regarding the importance of the Y chromosome structure in our manuscript. We have made revisions to enhance the discussion of the Y chromosome, including a comparative analysis of its structure between Mongolian cattle and the Y chromosomes of other bovine breeds (CM054900.2, CM037826.1, NC_082638.1, CP128563.1). This analysis aims to provide a clearer understanding of the Y chromosome's unique features (line129-132). We appreciate your insightful input and believe it has strengthened our manuscript.<br>2.Generally speaking, this manuscript didn't discover enough detail information or bright points. For example, the population genetic part was mostly enrichment analysis; it's a little rough and simple. We want to know the exactly gene and the specific mutation sites, which do associated with the formation of marbling patterns in beef.<br>Response：While we acknowledge that the population genetic analysis primarily focused on enrichment, we also recognize the need for a deeper exploration. Therefore, we will provide detailed information on the specific genes and mutation sites associated with the formation of marbling patterns in beef , which can be found in Table S9. By including these elements, we aim to strengthen the overall analysis and contribute valuable insights to the field.<br><br>Minor point：<br>1.The BUSCO of the telomere-to-telomere genome is 95.9%, which is good for chromosome-level genome, but not good enough for the telomere-to-telomere genome. The authors should explain this. Incidentally, the database of BUSCO that the author used for assessment should be showed in manuscript.<br>Response: Thank you for your feedback. The BUSCO score of 95.9% reflects the difficulty in assembling repetitive regions and complex structures, even in near telomere-to-telomere assemblies. We have now included the BUSCO dataset (mammalia_odb10) in the manuscript for clarity.<br>2.The genome quality assessment is quite important for T2T assembly, only BUSCO assessment is definitely not enough. Maybe the synteny alignment between Mongolian cattle and other species (not only Hereford cattle), the structure of MHC, or sex chromosomes structure is needed.<br>Response: Thank you for your valuable suggestions. We have now included genome quality assessments using QV analysis assessment to provide a more comprehensive evaluation of the assembly(line 126-128).<br>3.The description of population variant analysis (Line 153-156) is quite confused. How the non-reference sequences were obtained? How did these non-reference sequences mapped back to the cattle reference genome?<br>Response: Thank you for your valuable suggestion. We have clarified the description of the population variant analysis in lines 153-158. The analysis was performed using the PopIns2 software, and a detailed explanation of the methodology can now be found in lines 258-269, outlining how non-reference sequences were obtained and mapped back to the cattle reference genome.<br>4.Line 153: It's population, not population.<br>Response: We have carefully reviewed the spelling of 'population' and confirmed that it is correct in the manuscript. Please let us know if there is another aspect you were referring to.<br>5.The thousands separators are always missing.<br>Resopnse: Thank you for your feedback. We have now added the thousands separators where they were previously missing.
**Additional Information:**	
**Question**	**Response**
Are you submitting this manuscript to a special series or article collection?	No
**Experimental design and statistics**	Yes

Powered by Editorial Manager® and ProduXion Manager® from Aries Systems Corporation

Full details of the experimental design and statistical methods used should be given in the Methods section, as detailed in our Minimum Standards Reporting Checklist. Information essential to interpreting the data presented should be made available in the figure legends.<br><br>Have you included all the information requested in your manuscript?	
**Resources**<br><br>A description of all resources used, including antibodies, cell lines, animals and software tools, with enough information to allow them to be uniquely identified, should be included in the Methods section. Authors are strongly encouraged to cite Research Resource Identifiers (RRIDs) for antibodies, model organisms and tools, where possible.<br><br>Have you included the information requested as detailed in our Minimum Standards Reporting Checklist?	Yes
**Availability of data and materials**<br><br>All datasets and code on which the conclusions of the paper rely must be either included in your submission or deposited in publicly available repositories (where available and ethically appropriate), referencing such data using a unique identifier in the references and in the "Availability of Data and Materials" section of your manuscript.<br><br>Have you have met the above requirement as detailed in our Minimum Standards Reporting Checklist?	Yes

Manuscript
Click here to access/download;Manuscript;Manuscript-Mongolian Cattle-2024-9-25.doc

Near Telomere-to-Telomere Genome Assembly of Mongolian Cattle : Implications for Population Genetic Variation and Beef quality

Rina Su[1, *], Hao Zhou[2, *], Wenhao Yang[2, *], Sorgog Moqir[1], Xiji Ritu[1], Lei Liu[1],Ying Shi[1], Ai Dong[3], Menghe Bayier[4], Yibu Letu[5], Xin Manxi[5], Hasi Chulu[6], Narenhua Nasenochirb[7], He Meng[2, #], Muren Herrid[1, 8, #]

[1]Grassland & Cattle Investment Co., Ltd. Hohhot 010000, Inner Mongolia
[2]School of Agriculture and Biology, Shanghai Jiao Tong University, Shanghai 200240, China
[3]Bureau of Agriculture and Animal Husbandry, Alxa League, Bayanhot 750306, Inner Mongolia, China
[4]Centre for Animal Husbandry and Veterinary Technology, Alxa League, Bayanhot 750306, Inner Mongolia
[5] Station for Animal Husbandry, Xilingol League, Xilinhot 026000, Inner Mongolia
[6]Station for Animal Husbandry, Sunit Left Banner, Xilingol League, Xilinhot 026000, Inner Mongolia
[7]College of Animal Science, Inner Mongolia Agriculture University, Hohhot 010000, Inner Mongolia, China
[8]International Livestock Research Centre, Gold Coast, Queensland, Australia.

*Rina Su, Hao Zhou and Wenhao Yang contributed equally to this work.

[#]Corresponding author(s): He Meng (menghe@sjtu.edu.cn); Muren Herrid (mherrid@gmail.com)

Abstract

Background

Mongolian cattle, a unique breed indigenous to China, represent valuable genetic resources and serve as important sources of meat and milk. However, there is a lack of high-quality genomes in cattle, which limits biological research and breeding improvement.

Findings

In this study, we conducted whole-genome sequencing on a Mongolian bull. This effort yielded a 3.1 Gb Mongolian cattle genome sequence, with a BUSCO integrity assessment of 95.9%. The assembly achieved both Contig N50 and Scaffold N50 values of 110.9 Mb, with only 3 gaps identified across the entire genome. Additionally, We successfully assembled the Y chromosome among the 31 chromosomes. Notably, 3 chromosomes were identified as having telomeres at both ends. The annotation data includes 54.31% repetitive sequences, 29,794 coding genes. Furthermore, a population genetic variation anlysis was conducted on 332 individuals from 56 breeds, through which we identified variant loci and potentially discovered genes associated with the formation of marbling patterns in beef, predominantly located on chromosome 12.

Conclusions

This study produced a genome with high continuity, completeness, and accuracy, marking the first assembly and annotation of a near telomere-to-telomere genome in cattle. Based on this, we generated a variant database comprising 332 individuals. The assembly of the genome and the analysis of population variants provide significant insights into cattle evolution and enhance our understanding of breeding selection.

Keywords: Mongolian Cattle, Near Telomere-to-Telomere Genome, Beef Quality, Population Genetic Variation

Background

The Mongolian cattle originated in the Mongolian Plateau and are now distributed in regions such as Inner Mongolia, Heilongjiang, and Hebei[1]. They are an excellent breed of cattle (Bos taurus) in China and a valuable genetic resource for the China[2]. In the Inner Mongolia region, which features high altitudes and dry weather, Mongolian cattle have developed strong resistance to cold, drought, and adverse conditions through long-term adaptive selection[3]. Despite the significant role of Mongolian cattle in China's cattle industry, genomic information about this breed is relatively scarce.

With the advancement of third-generation single-molecule sequencing technology, genomic research has progressed rapidly. Currently, organisms such as humans, rice and others have successively achieved telomere-to-telomere (T2T) level reference genome assembly[4, 5]. The updated genome versions not only offer more comprehensive information on genome sequences and variation maps but also valuable insights into previously challenging "genomic desert" areas, including telomeres, centromeres, and repeat sequence regions. These regions have been identified as crucial for the development of species-specific diseases and the formation of phenotypes[6].

As important economic animals, cattle contribute significantly to agriculture worldwide. Their genetic enhancement is directly correlated with advancements in genomic research. Nevertheless, current bovine reference genomes, primarily derived from European cattle breeds, suffer from several limitations, such as incomplete assemblies and identified gaps[7-9]. These deficiencies underscore the imperative for a more comprehensive and precise bovine genome assembly to inform biological studies and breeding programs, particularly for non-European cattle breeds. Currently, there is limited genomic assembly information available for Chinese indigenous cattle, leading to an incomplete understanding of their genomic characteristics. Additionally, the genetic mechanisms of cattle traits, such as meat quality, also need to be studied.

Therefore, to address the lack in the genomic research of indigenous Chinese cattle, we utilized a combination of next-generation and third-generation sequencing

technologies to assemble a near telomere-to-telomere genome of the Mongolian cattle. Furthermore, utilizing the Mongolian assembly as a reference, we additionally resequenced 95 individuals and downloaded data for 237 individuals from public databases for population variation analysis. By comparing 56 breeds, we identified 106 million SNPs, 4.89 million insertions, and 5.4 million deletions, which provided us with a comprehensive understanding of the cattle population genome. It is interesting to note that, upon comparing the genomes of other cattle and Wagyu cattle, potential genes associated with the formation of beef marbling patterns were identified. The genome of indigenous Chinese cattle provides valuable insights into the genetic basis and population structure of native cattle breeds. This knowledge not only enhances the selection and breeding practices of breeders but also plays a pivotal role in the conservation efforts of local Chinese breeds.

Results

Construction of High-quality Sequence Maps

We collected a total of 114 Gb HiFi data, 155 Gb ONT data (N50>75 Kb), and 403.6 Gb Hi-C data (Table S1). The assembly was conducted using Hifiasm[10]. After joint assembly and redundancy removal, we obtained the final contig assembly (Table S2). This version comprises of 56 contigs with a total length of 3.1Gb and a Contig N50 of 110.9 Mb. Following auxiliary assembly using Hi-C data, 53 sequences were generated. Visualization based on scaffold interaction intensity and position revealed distinct groupings on the Hi-C heatmap (Figure 1B). Within each grouping, interaction strength at the diagonal exceeded that at non-diagonal positions, indicating effective genome anchoring through Hi-C assistance. Notably, ptg000040l, ptg000060l, and ptg000025l assembled into the X chromosome, while ptg000039l and ptg000030l formed a chromosome. Further analysis confirmed 28 contigs fully matching cattle reference genome chromosomes (Figure 2A). The remaining 23 contigs were inferred to belong to the Y chromosome. By aligning and assembling these contigs with the scaffold of the male cattle Y chromosome(CP128563.1), 3

contigs were assembled into the Mongolian cattle Y chromosome, resulting in a Y chromosome length of 17.9Mb. At this point, 5 gaps remained in the genome. Gap filling addressed 1 gap in chromosome 6 and 1 gap in the chromosome X, leaving 3 gaps unresolved. The final genome version post gap filling was defined as the definitive version used for subsequent analyses (Table S3).

The Contig N50 of the Mongolian cattle genome significantly surpassed those of published Hereford cattle genomes (Table 1). Assessment using BUSCO software indicated the assembled Mongolian cattle genome's completeness at 95.9% (Figure 2B), underscoring its high quality. We also assessed accuracy and completeness using Merqury[11], which yielded an assembly QV value of 54.732 and a completeness of 95.223% (Table S4, Figure S1).

Due to its complex structure, the Y chromosome has consistently posed challenges for sequencing and assembly. We conducted a synteny analysis of the assembled Y chromosome with other assembled versions, revealing high consistency and homology (Figure S2). Upon sequence identification of assembled scaffolds, telomeres were identified at one end of 23 chromosomes. Notably, telomeric sequences were observed at both ends of the chromosome X, chromosome 21, and chromosome 25 (Table S3), suggesting potential T2T level assembly for these three chromosomes. We also used Centromics[12] and quarTeT[13] CentroMiner to identify centromeres by detecting high-copy tandem repeats, selecting candidate sites for the centromeres (Figure S3).

Genome Annotation Information

Repeated sequences in the Mongolian cattle genome include dispersed repeats and tandem repeats. These sequences, classified as LTR (long terminal repeat), LINE (long interspersed nuclear element), SINE (short interspersed element), and DNA transposons, collectively account for 54.31% of the genome, consistent with patterns observed in mammals, validating the accuracy of repeat identification (Table 2). Following the masking of repetitive sequences, Liftoff was employed for annotation, revealing a total of 29,794 protein-coding genes (Figure 2C).

This extensive catalog of protein-coding genes forms a critical basis for understanding genome functionality. Additionally, non-coding RNAs (ncRNAs) were identified, including 1,082 transfer RNAs (tRNAs), 955 small nuclear RNAs (snRNAs), 612 small nucleolar RNAs (snoRNAs), and 7,240 long non-coding RNAs (lncRNAs), underscoring their roles in gene regulation and epigenetic mechanisms. This comprehensive annotation sheds light on the intricate genomic architecture and sets the stage for future functional and comparative genomic investigations.

Construction Cattle Genetic Variation Database

In this study, we sequenced 95 individuals from 11 cattle breeds, obtaining a total of 4.17 Tb of base data (Table S5). Additionally, we collected data for 237 individuals from 45 breeds from the NCBI SRA database(Table S6). Using our assembled genome as the reference, we aligned the data from 56 breeds and identified 106 million SNPs, 4.89 million insertions, and 5.4 million deletions to form a comprehensive cattle population genetic variation database (Figure 3).

Using PopIns2 for population variant analysis, we identified a total of 674 Mb of novel non-reference sequences from 332 cattle individuals. The longest of these sequences was 1,280,529 bp, and the assembly had an N50 value of 29,590 bp. After aligning these sequences back to the cattle reference genome, we identified 2,845 SVs. These SVs represent potentially important genetic variations that are absent from the reference genome and may contribute to breed-specific traits in cattle populations.

Comparative Genomics Analysis

Despite the unique characteristics exhibited by certain cattle breeds, the underlying genetic mechanisms remain largely unknown. Wagyu cattle, renowned for their heavily marbled meat, offer superior tenderness, flavor, and juiciness, distinguishing them in the global beef market[14]. To explore genomic differences between Mongolian cattle and Wagyu, we conducted a comparative genomics analysis using the assembled Mongolian cattle genome. We identified a total of 99,429,985 common variant sites and computed Fst values (Figure 4A). From these, 994,299 candidate sites within 735,339 genes were selected for further analysis. Functional and pathway enrichment analysis revealed 43 significantly enriched functions, including cell

junction, lipid binding, long-chain fatty acid transport, and developmental growth involved in morphogenesis. Additionally, we identified 145 enriched pathways such as Axon guidance, Calcium signaling pathway, B cell receptor signaling pathway, and Growth hormone synthesis, secretion, and action (Table S7).

Over years of artificial selection, Wagyu beef has developed a distinct marbling pattern. To decipher the mechanisms behind its exceptional meat quality, we conducted comparative genomics analysis between Wagyu and other beef cattle (Figure 4B, Table S6). Initially, we computed FST values and discovered 95010853 total variants. After screening, we decided on 954652 differential variant sites using a threshold of 0.422786. Annotation analysis revealed 18976 candidate genes. Functional annotation of these genes found 12 significantly enriched functions such as proteolysis, lipid binding, postsynapse, secretory granule membrane. Furthermore, 184 enriched pathways were identified, including Axon guidance, Focal adhesion, and cAMP signaling pathway (Table S8).

In addition, we performed Genome-Wide Association Study (GWAS) analysis on the beef cattle population to further select relevant sites (Figure 4C, Table S9). This analysis identified 54,250 significant sites using both GLM (Generalized Linear Model) and MLM (Mixed Linear Model) models. Notably, a significant signal was detected on chromosome 12 of the Wagyu genome within specific intervals. This region encompasses eight genes: FAM155A, KLF12, KLHL1, PCDH9, ATP12A, MPHOSPH8, LOC101902228, and RFC3, potentially playing crucial roles in the formation of the marbled pattern in Wagyu beef (Table S10).

Method

Sample Collection and Sequencing

This study conducted whole-genome sequencing using blood samples from a five-year-old Mongolian cattle in the Xilingol region. The collected samples were stored at -80°C until DNA extraction. The collection and handling of these samples were carried out in accordance with approved guidelines and regulations from Shanghai Jiao Tong University.

Library construction was carried out in accordance with the official recommendations of various sequencing platforms. The PacBio Sequel IIe offers high quality long read sequences(HiFi reads)[15]. PromethION P48 generates Ultralong Oxford Nanopore Reads(ONT reads)[16]. Hi-C(Chromosome conformation capture) sequencing was generates from novaseq 6000 [17]. In total, we collected 114 Gb of HiFi data, 155 Gb of ONT data (with N50 > 75 Kb), and 403.6 Gb of Hi-C data (Table S1). Second-generation sequencing were obtained from the Illumina NovaSeq 6000 instrument. Besides, 95 individuals generated in this study, which were resequenced using second-generation sequencing technology at the BGI platform(Table S5). An additional 237 individuals had their genome sequences obtained from the NCBI SRA database (Table S6).

Genome Assembly

We utilized Hifiasm v0.16.1-r375 for genome assembly, leveraging its novel approach to construct ultralong-read overlapped graphs[10]. Initially, error-prone long reads were mapped against themselves to form an initial graph, which was then iteratively simplified by trimming tips and resolving bubbles to achieve the final assembly. This method has demonstrated efficacy in producing high-quality assemblies with substantial contig N50 values. Throughout our study, assembly parameters were meticulously adjusted to optimize genome quality based on N50 and gap count, using default settings in the software package.

Furthermore, purge_dups v1.2.5[18] was employed to eliminate redundant heterozygous duplications, which can significantly impact assembly accuracy. This algorithm utilizes read depth information and sequence similarity to identify and remove redundant contigs, thereby improving assembly fidelity.

To scaffold the genome, we employed Chromap v0.2.5-r473 [19] and yahs v 1.2 [20] software suites in conjunction with Hi-C data. Chromap efficiently maps high-throughput chromatin conformation capture (Hi-C) data and integrates it into the assembly process. Yahs utilizes Hi-C interaction frequencies to correct misassemblies and organize assembled sequences into clusters, ensuring accurate orientation and order. For assembling the Y chromosome, the existing cattle genome Y chromosome

sequence from NCBI (CP128563.1) served as the reference scaffold, and the assembly was further refined using RagTag[21]. Quality assessment of the assembled genome was conducted using BUSCO v5.4.4[22] (mammalia_odb10) for evaluating gene space completeness and Quast v5.1.0rc1[23] for analyzing key genomic metrics such as GC content and total length. We used Merqury[11], a k-mer-based genome assessment tool, to evaluate the accuracy and completeness of this assembly. First, we created a database of the second-generation sequencing data using Meryl, followed by running Merqury for the analysis.

We downloaded four Y chromosome assemblies from NCBI: CM054900.2, CM037826.1, NC_082638.1, and CP128563.1. These assemblies were used to perform synteny analysis with the newly assembled Y chromosome of Mongolian cattle. For this analysis, we utilized NGenomeSyn[24], a synteny visualization tool that leverages the alignment capabilities of Minimap2 to detect and display syntenic blocks between genomes.

Telomere and centromeres Identification

We used quarTeT v1.1.5 TeloExplorer to identify telomeres and CentroMiner to identify centromeres[13]. TeloExplorer detects canonical vertebrate telomere "TTAGGG" repeats across contigs, while CentroMiner identifies high-copy tandem repeats typical of centromeric regions. Due to the complex structure of centromeres, we also used Centromics to identify centromeres by detecting high-copy tandem repeats from PacBio sequencing data.

Annotation

We employed RepeatMasker v2.0.2[25] for detecting and masking repetitive elements within the genome, utilizing the Repbase database, the most comprehensive source of repetitive element annotations. RepeatMasker provides detailed annotations of the locations and classifications of repetitive DNAs.

Following this, Liftoff v1.6.3[26] was utilized to transfer annotations between genomes with discrepancies, annotating protein-coding genes, long non-coding RNAs (lncRNAs), and small RNAs in the masked assembly. We utilized the Bos taurus reference genome (fa file) and its gene annotation (gff file) from the NCBI database

for this purpose.

Variant Calling

The dataset included 95 individuals generated in this study, which were resequenced using second-generation sequencing technology at BGI, and 237 individuals whose genome sequences were obtained from public databases. The 332 clean data were aligned to our assembled reference genome using BWA MEM 0.7.17-r1188[27], a software that utilizes Burrows-Wheeler Transform to perform rapid and precise alignment. Following this, GATK 4.3.0.0 suite[28] was employed for variant calling. The GATK pipeline includes four main steps: base quality score recalibration (BQSR), indel realignment, duplicate removal, and variant calling. Initially, base quality scores are recalibrated to minimize machine-specific errors. Afterward, the local realignment step is done around indels to correct misalignments due to the presence of indels. Next, potential PCR duplicates are removed. Finally, the resulting cleaned, recalibrated reads are used for variant calling.

The analysis was performed using popins2 v0.13.0[29] software suite on 332 cattle from various populations. The popins2 software provides a computational pipeline for discovering and genotyping novel sequence insertions in many individuals simultaneously. Initially, reads were aligned to the reference genome with BWA MEM to generate BAM files. Then, a population assembly of sequences not present in the reference genome was created using FermiKit v0.13[30]. Contigs across all individuals were merged using Minimus2[31] into a single FASTA file. Popins2 subsequently aligned these contigs back to the reference genome and called insertion sites. Genotyping of insertion polymorphisms was performed in all individuals using a likelihood model implemented in popins2, which utilizes counts of reads supporting both the reference and insertion alleles. Finally, low-quality insertion genotypes and variants were filtered based on various quality metrics provided by the software.

Comparative Genomic Analysis

Based on the obtained genetic variation dataset, comparative genomic analyses were performed. Measures of population differentiation, Fst, were computed using VCFtools 0.1.16[32]. SnpEff v48.0 [33] was deployed for predicting the effects of

identified variants while GO and KEGG pathway analyses were conducted using the R package clusterProfiler v4.0 [34]. For deeper insights into the distinct marbled beef characteristic of Wagyu cattle, Genome-Wide Association Studies (GWAS) were performed using FarmCPU v1.02[35], an R package for multiple locus mapping.

Discussion

With the introduction of third-generation single-molecule sequencing technology, we have achieved substantial advancements in bovine genome assembly. Our assembled Mongolian cattle genome exhibits a high quality with a Contig N50 of 110.9 Mb and only 3 remaining gaps. Previous studies predominantly relied on European cattle reference genomes, potentially introducing biases in the analysis of Asian cattle data[36]. Our study not only presents an improved bovine genome assembly characterized by fewer gaps compared to previous assemblies, but it also greatly contributes to future genetic research on Asian cattle breeds. Furthermore, our insights into telomeres and centromeres, crucial elements for chromosomal stability and cell division, add valuable knowledge to this limited research field.

Existing cattle genetic variation databases are pivotal for investigating population genetics, breed improvements, and disease resistance. Our comprehensive database, comprising data from 56 cattle breeds worldwide, contains 106 million SNPs, establishing it as the most extensive bovine genetic variation database to date. Our high-quality assembled genome helped us identify an even greater number of non-reference sequences (674 Mb), thereby providing a more precise representation of the bovine genomic diversity.

The distinctive marbling traits of Wagyu beef are highly prized, yet their genetic underpinnings remain poorly understood [14]. Through our updated genome assembly, we identified significant genetic signals harboring genes like FAM155A, KLF12, KLHL1, PCDH9, ATP12A, MPHOSPH8, LOC101902228, and RFC3. Notably, PCDH9 influences lipid metabolism,potentially impacting fat deposition and marbling in beef[37]. Similarly, ATP12A is involved in muscle pH regulation, affecting meat

quality[38]. KLHL1 is associated with skeletal muscle development, possibly impacting cattle growth[39]. Explorin g these genes promises valuable insights into the genetic basis of beef quality, facilitating targeted breeding and management strategies.

In conclusion, this study marks a significant advancement in bovine genomics by providing a high-quality reference genome for Mongolian cattle and shedding light on crucial genetic elements such as telomeres and centromeres. By overcoming gaps and biases associated with previous European-centric studies, our findings pave the way for more precise genetic research and breeding programs tailored to Asian cattle breeds. Furthermore, the identification of key genes associated with Wagyu beef marbling, including PCDH9, ATP12A, and KLHL1, underscores their potential roles in shaping meat quality traits. These insights not only enhance our understanding of beef production but also offer actionable knowledge for improving genetic selection strategies in cattle breeding worldwide

Authors' Contributions

R.S., H.Z. and W.Y. contributed equally to this work. H.Z., W.Y., H.M., M.H. contributed to the study's conception and design, and M.H is the principal investigator. R.S., N.N., S.M., X.R., L.L., Y.S., A.D., M.B., Y.L., X.M., H.C., and N.N. were involved in material preparation, data collection, and DNA extraction. R.S., H.Z., W.Y., H.M., and M.H. evaluated the study's quality. H.Z., W.Y., H.M., and M.H. wrote and edited the manuscript. All authors read and approved the final manuscript.

Data Availability

The whole genome sequence data reported in this paper have been deposited in the Genome Warehouse[40] in National Genomics Data Center[41], Beijing Institute of Genomics, Chinese Academy of Sciences / China National Center for Bioinformation, under BioProject number PRJCA023030 and accession number GWHERBZ00000000.1 that is publicly accessible at https://ngdc.cncb.ac.cn/gwh.
The variation data reported in this paper has been deposited in the Genome Variation Map[42] in National Genomics Data Center[41], China National Center for Bioinformation / Beijing Institute of Genomics, Chinese Academy of Sciences, under accession number GVM000798 that can be publicly accessible at http://bigd.big.ac.cn/gvm/getProjectDetail?project=GVM000798.
The raw sequence data reported in this paper have been deposited in the Genome Sequence Archive[43] in National Genomics Data Center[41], China National Center for Bioinformation / Beijing Institute of Genomics, Chinese Academy of Sciences (GSA: CRA017637) that are publicly accessible at https://ngdc.cncb.ac.cn/gsa.

Competing Interests

The authors declare no competing interests.

Funding

This research was funded by the Department of Science and Technology of the Inner Mongolia Autonomous Region, China, under the "Revelation and Leadership" project (Project number: 2022JBGS0023).

References:

1. Chen Q. *et al.*, Whole genome analyses revealed genomic difference between European taurine and East Asian taurine. *Journal of Animal Breeding and Genetics* **138**, 56-68 (2021).
2. Fedotova G. V., Slozhenkina M. I., Tsitsige, Natyrov A. K., Erendzhenova M. V., Comparative analysis of economic and biological features of Kalmyk and Mongolian cattle breeds. *IOP Conference Series: Earth and Environmental Science* **548**, 082076 (2020).
3. Ahmad A. A. *et al.*, Age-dependent variations in rumen bacterial community of Mongolian cattle from weaning to adulthood. *BMC Microbiol* **22**, 213 (2022).
4. Nurk S. *et al.*, The complete sequence of a human genome. *Science* **376**, 44-53 (2022).
5. Shang L. *et al.*, A complete assembly of the rice Nipponbare reference genome. *Mol Plant* **16**, 1232-1236 (2023).
6. Sedlazeck F. J., Lee H., Darby C. A., Schatz M. C., Piercing the dark matter: bioinformatics of long-range sequencing and mapping. *Nat Rev Genet* **19**, 329-346 (2018).
7. Zimin A. V. *et al.*, A whole-genome assembly of the domestic cow, Bos taurus. *Genome Biol* **10**, R42 (2009).
8. Li T.-T. *et al.*, De novo genome assembly depicts the immune genomic characteristics of cattle. *Nature Communications* **14**, 6601 (2023).
9. Jang J. *et al.*, Chromosome-level genome assembly of Korean native cattle and pangenome graph of 14 Bos taurus assemblies. *Sci Data* **10**, 560 (2023).
10. Cheng H., Concepcion G. T., Feng X., Zhang H., Li H., Haplotype-resolved de novo assembly using phased assembly graphs with hifiasm. *Nat Methods* **18**, 170-175 (2021).
11. Rhie A., Walenz B. P., Koren S., Phillippy A. M., Merqury: reference-free quality, completeness, and phasing assessment for genome assemblies. *Genome Biology* **21**, 245 (2020).
12. https://github.com/zhangrengang/Centromics/tree/master/Centromics
13. Lin Y. *et al.*, quarTeT: a telomere-to-telomere toolkit for gap-free genome assembly and centromeric repeat identification. *Hortic Res* **10**, uhad127 (2023).
14. Gotoh T. *et al.*, Differences in muscle and fat accretion in Japanese Black and European cattle. *Meat Sci* **82**, 300-308 (2009).
15. Wenger A. M. *et al.*, Accurate circular consensus long-read sequencing improves variant detection and assembly of a human genome. *Nature Biotechnology* **37**, 1155-1162 (2019).
16. Wang Y., Zhao Y., Bollas A., Wang Y., Au K. F., Nanopore sequencing technology, bioinformatics and applications. *Nature Biotechnology* **39**, 1348-1365 (2021).
17. Belton J. M. *et al.*, Hi-C: a comprehensive technique to capture the conformation of genomes. *Methods* **58**, 268-276 (2012).
18. Guan D. *et al.*, Identifying and removing haplotypic duplication in primary genome assemblies. *Bioinformatics* **36**, 2896-2898 (2020).

19. Zhang H. *et al.*, Fast alignment and preprocessing of chromatin profiles with Chromap. *Nature Communications* **12**, 6566 (2021).
20. Zhou C., McCarthy S. A., Durbin R., YaHS: yet another Hi-C scaffolding tool. *Bioinformatics* **39**, (2022).
21. Alonge M. *et al.*, Automated assembly scaffolding using RagTag elevates a new tomato system for high-throughput genome editing. *Genome Biology* **23**, 258 (2022).
22. Seppey M., Manni M., Zdobnov E. M., BUSCO: Assessing Genome Assembly and Annotation Completeness. *Methods Mol Biol* **1962**, 227-245 (2019).
23. Gurevich A., Saveliev V., Vyahhi N., Tesler G., QUAST: quality assessment tool for genome assemblies. *Bioinformatics* **29**, 1072-1075 (2013).
24. He W. *et al.*, NGenomeSyn: an easy-to-use and flexible tool for publication-ready visualization of syntenic relationships across multiple genomes. *Bioinformatics* **39**, (2023).
25. Tarailo-Graovac M., Chen N., Using RepeatMasker to identify repetitive elements in genomic sequences. *Curr Protoc Bioinformatics* **Chapter 4**, 4.10.11-14.10.14 (2009).
26. Shumate A., Salzberg S. L., Liftoff: accurate mapping of gene annotations. *Bioinformatics* **37**, 1639-1643 (2021).
27. Jung Y., Han D., BWA-MEME: BWA-MEM emulated with a machine learning approach. *Bioinformatics* **38**, 2404-2413 (2022).
28. Brouard J. S., Bissonnette N., Variant Calling from RNA-seq Data Using the GATK Joint Genotyping Workflow. *Methods Mol Biol* **2493**, 205-233 (2022).
29. Krannich T. *et al.*, Population-scale detection of non-reference sequence variants using colored de Bruijn graphs. *Bioinformatics* **38**, 604-611 (2021).
30. Li H., FermiKit: assembly-based variant calling for Illumina resequencing data. *Bioinformatics* **31**, 3694-3696 (2015).
31. Sommer D. D., Delcher A. L., Salzberg S. L., Pop M., Minimus: a fast, lightweight genome assembler. *BMC Bioinformatics* **8**, 64 (2007).
32. Danecek P. *et al.*, The variant call format and VCFtools. *Bioinformatics* **27**, 2156-2158 (2011).
33. Cingolani P. *et al.*, A program for annotating and predicting the effects of single nucleotide polymorphisms, SnpEff: SNPs in the genome of Drosophila melanogaster strain w1118; iso-2; iso-3. *Fly (Austin)* **6**, 80-92 (2012).
34. Yu G., Wang L. G., Han Y., He Q. Y., clusterProfiler: an R package for comparing biological themes among gene clusters. *Omics* **16**, 284-287 (2012).
35. Liu X., Yin L., Zhang H., Li X., Zhao S., Performing Genome-Wide Association Studies Using rMVP. *Methods Mol Biol* **2481**, 219-245 (2022).
36. Talenti A. *et al.*, A cattle graph genome incorporating global breed diversity. *Nature Communications* **13**, 910 (2022).
37. Lu X. *et al.*, Genome-Wide Association Study on Reproduction-Related Body-Shape Traits of Chinese Holstein Cows. *Animals (Basel)* **11**, (2021).
38. Kim G.-D. *et al.*, The influence of fiber size distribution of type IIB on carcass traits and meat quality in pigs. *Meat Science* **94**, 267-273 (2013).

39. Aromolaran K. A., Benzow K. A., Cribbs L. L., Koob M. D., Piedras-Rentería E. S., T-type current modulation by the actin-binding protein Kelch-like 1. *Am J Physiol Cell Physiol* **298**, C1353-1362 (2010).
40. Chen M. *et al.*, Genome Warehouse: A Public Repository Housing Genome-scale Data. *Genomics Proteomics Bioinformatics* **19**, 584-589 (2021).
41. Database Resources of the National Genomics Data Center, China National Center for Bioinformation in 2024. *Nucleic Acids Res* **52**, D18-d32 (2024).
42. Li C. *et al.*, Genome Variation Map: a worldwide collection of genome variations across multiple species. *Nucleic Acids Res* **49**, D1186-d1191 (2021).
43. Chen T. *et al.*, The Genome Sequence Archive Family: Toward Explosive Data Growth and Diverse Data Types. *Genomics Proteomics Bioinformatics*, (2021).

Figure Legend

Figure 1: (A). Morphological photograph of Mongolian cattle **(B).** Hi-C chromatin interaction map of the Mongolian cattle assembly, with chromosomes presented from top to bottom and from left to right, representing Chr1-Chr29, ChrX.

Figure 2: (A). The pairwise genome alignments of the Mongolian genome and the Hereford cattle genome are displayed. (**B**) Bar chart illustrating the BUSCO assessment of the Mongolian cattle genome. (**C**) The circos plot of the Mongolian cattle genome assembly. The rings from outside to inside indicate (a) chromosomes of the Mongolian genome, (b) GC density, (c) Gene densit and (d) Repeat density, b-d were drawn in 100kb sliding windows.

Figure 3: (A) Bar chart depicting the number and proportion of various variations in cattle. Among them, nSNPs account for 101,612,545 (88%), nInsertions for 4,899,369 (4.2%), nDeletions for 5,408,006 (4.7%), nComplex for 1,532,156 (1.3%), and nMixed for 1,980,238 (1.7%), with the remainder being zero. **(B)** Distribution of SNPs on chromosomes

Figure 4 : (A) Manhattan plot of the variant sites from the comparative analysis between Mongolian cattle and Wagyu. **(B)** Manhattan plot of the variant sites the comparative analysis between Wagyu and other beef cattle. **(C)** GWAS analysis of beef cattle populations using GLM and MLM models.

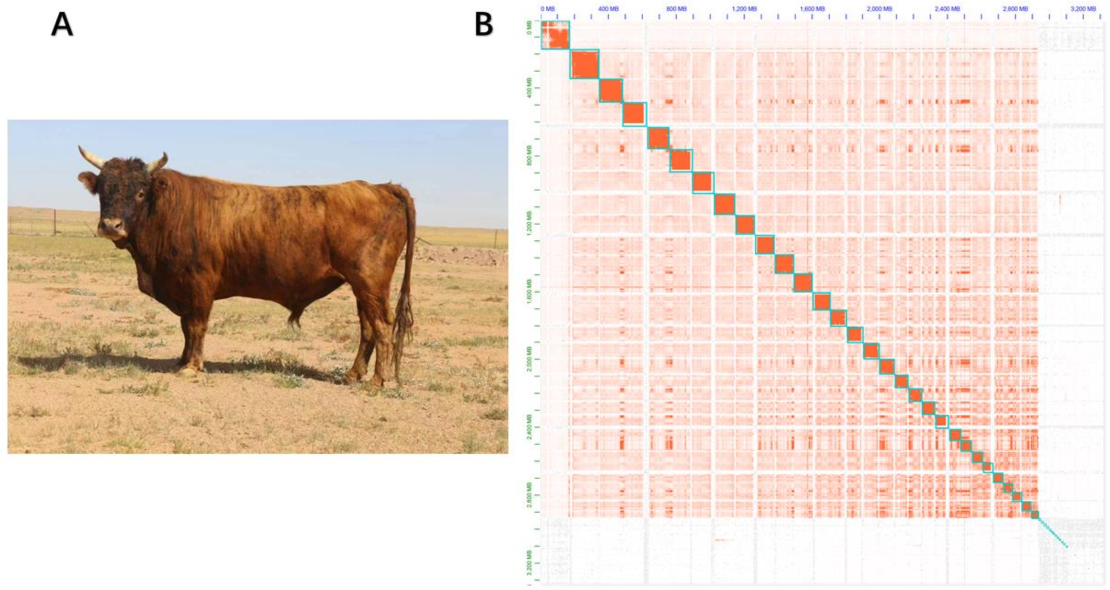
A
B
0 MB
400 MB
800 MB
1,200 MB
1,600 MB
2,000 MB
2,400 MB
2,800 MB
3,200 MB

Figure1

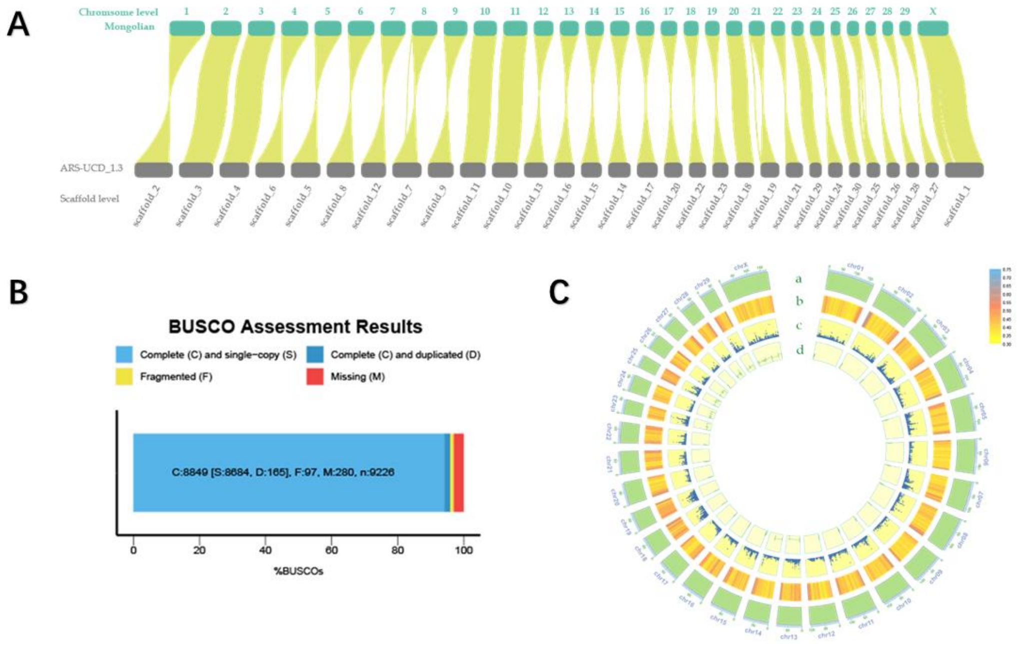
A
Chromsome level
Mongolian
1 2 3 4 5 6 7 8 9 10 11 12 13 14 15 16 17 18 19 20 21 22 23 24 25 26 27 28 29 X
ARS-UCD_1.3
Scaffold level
scaffold_2 scaffold_3 scaffold_4 scaffold_6 scaffold_5 scaffold_8 scaffold_12 scaffold_7 scaffold_9 scaffold_11 scaffold_10 scaffold_13 scaffold_16 scaffold_15 scaffold_14 scaffold_17 scaffold_20 scaffold_22 scaffold_23 scaffold_18 scaffold_19 scaffold_21 scaffold_29 scaffold_24 scaffold_30 scaffold_25 scaffold_26 scaffold_28 scaffold_27 scaffold_1
B
BUSCO Assessment Results
Complete (C) and single-copy (S)
Complete (C) and duplicated (D)
Fragmented (F)
Missing (M)
C:8849 [S:8684, D:165], F:97, M:280, n:9226
0 20 40 60 80 100
%BUSCOs
C
a
b
c
d

Figure2

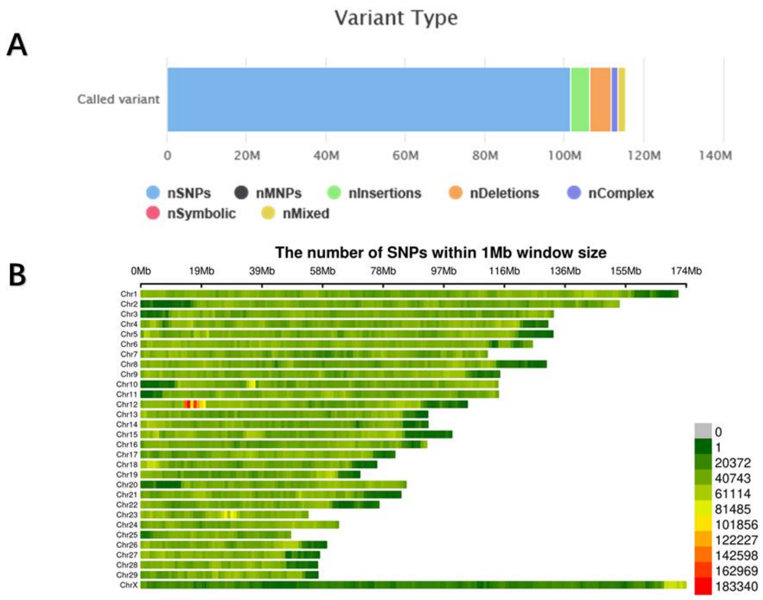
A
Variant Type
Called variant
0
20M
40M
60M
80M
100M
120M
140M
nSNPs
nMNPs
nInsertions
nDeletions
nComplex
nSymbolic
nMixed
B
The number of SNPs within 1Mb window size
0Mb
19Mb
39Mb
58Mb
78Mb
97Mb
116Mb
136Mb
155Mb
174Mb
0
1
20372
40743
61114
81485
101856
122227
142598
162969
183340

Figure3

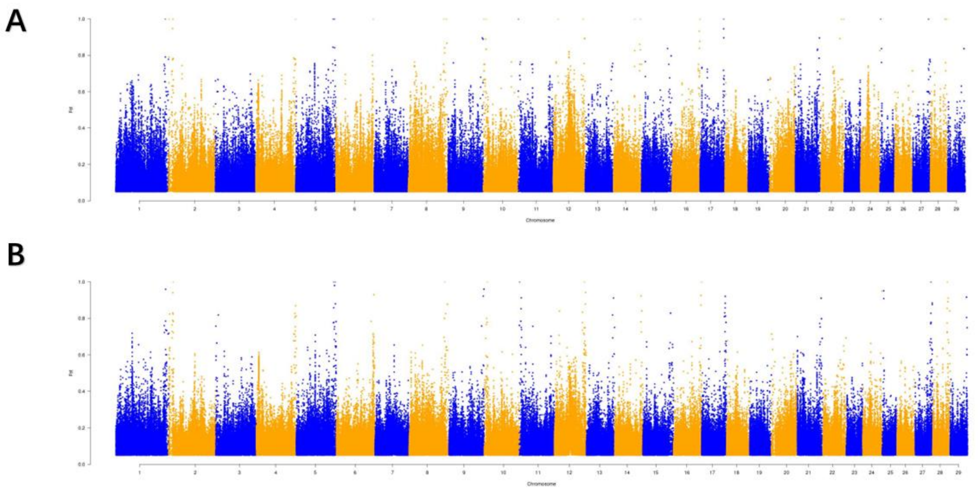

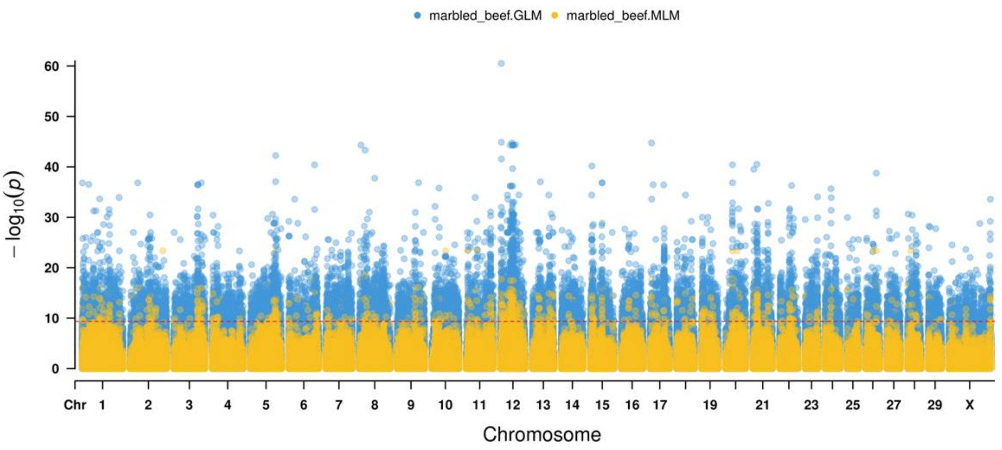

Figure4

Table 1 Genome assembly statistics

Genomic features	ARS-UCD1.3	Mongolia v1.3
Total length (Gp)	2.7 Gb	3.1 Gb
Number of contigs	2342	56
Number of scaffolds	1956	53
Contig N50 (bp)	25.9	110.9
Scaffold N50 (Mb)	103.3	110.9
Longest contig (bp)	119,708,465	171,934,863
Longest scaffold (bp)	158,534,110	174,471,981
GC content	41.5	43.41
Number of chromosomes	30	31

Table 2 Statistics of repetitive elements

Element Category	Element	Number of Elements	Length Occupied(bp)	Percentage of Sequence
SINEs		2,117,318	317,605,131	10.23%
	MIRs	403,380	58,075,101	1.87%
LINEs:		1,347,248	760,478,313	24.49%
	LINE1	598,858	349,393,159	11.25%
	LINE2	259,858	67,070,932	2.16%
	L3/CR1	34,951	7,232,125	0.23%
	RTE	452,385	336,604,273	10.84%
LTR elements:		474,297	163,941,172	5.28%
	ERVL	76,222	30,060,567	0.97%
	ERVL-MaLRs	122,838	40,272,757	1.30%
	ERV_classI	92,748	40,285,943	1.30%
	ERV_classII	165,418	49,313,737	1.59%
DNA elements:		293,384	58,196,559	1.87%
	hAT-Charlie	165,710	30,826,106	0.99%
	TcMar-Tigger	45,550	12,079,090	0.39%
Unclassified:		3,032	466,630	0.02%
Total interspersed repeats:			1,300,687,805	41.89%
Small RNA:	-	260,501	4,4038,646	1.42%
Satellites:	-	53,009	356,125,180	11.47%
Simple repeats:	-	570,811	24,190,888	0.78%
Low complexity:	-	85,741	4,213,890	0.14%

Click here to access/download;Figure;figure1.png

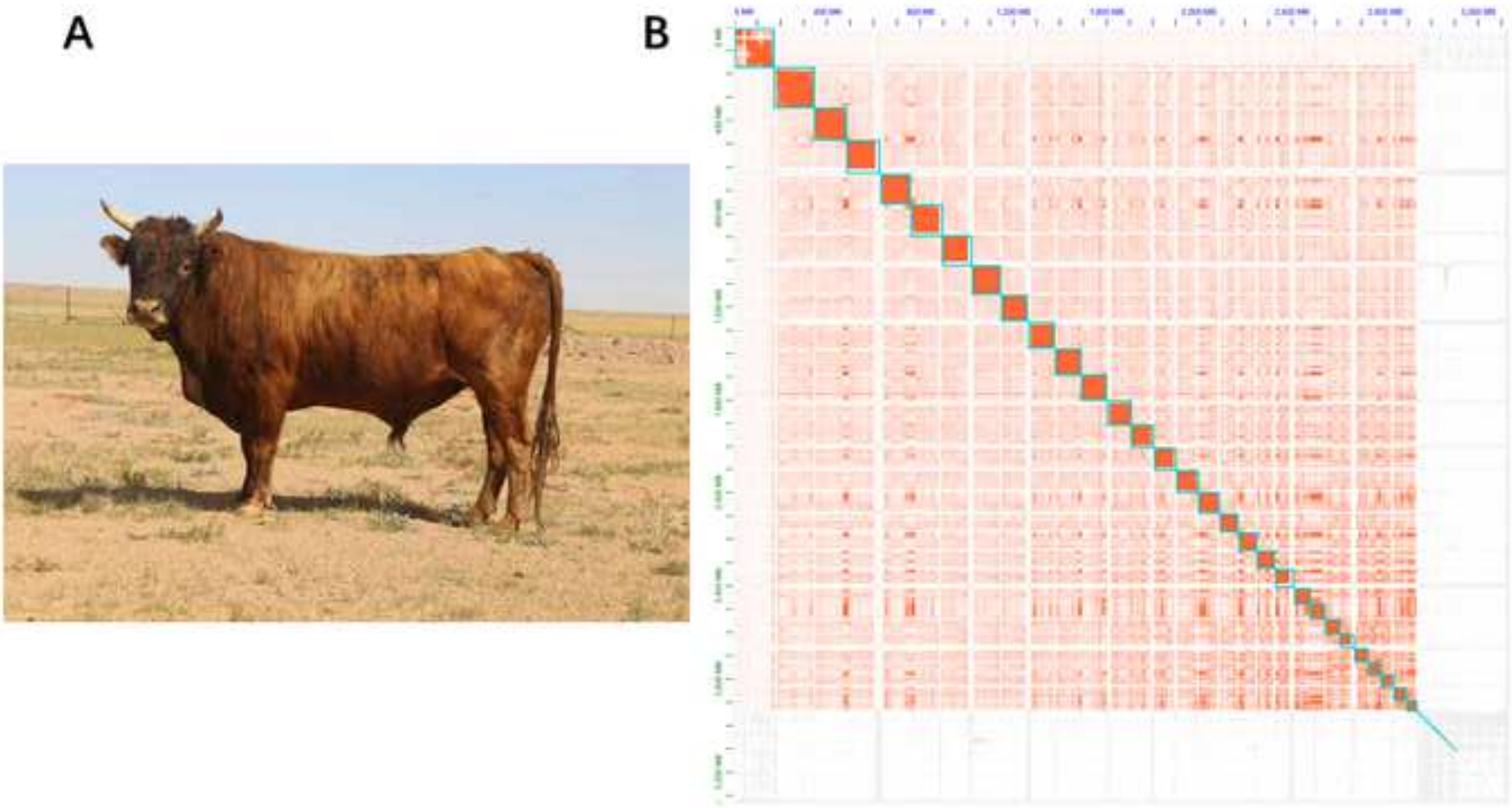

Click here to access/download;Figure;Figure1A.jpg

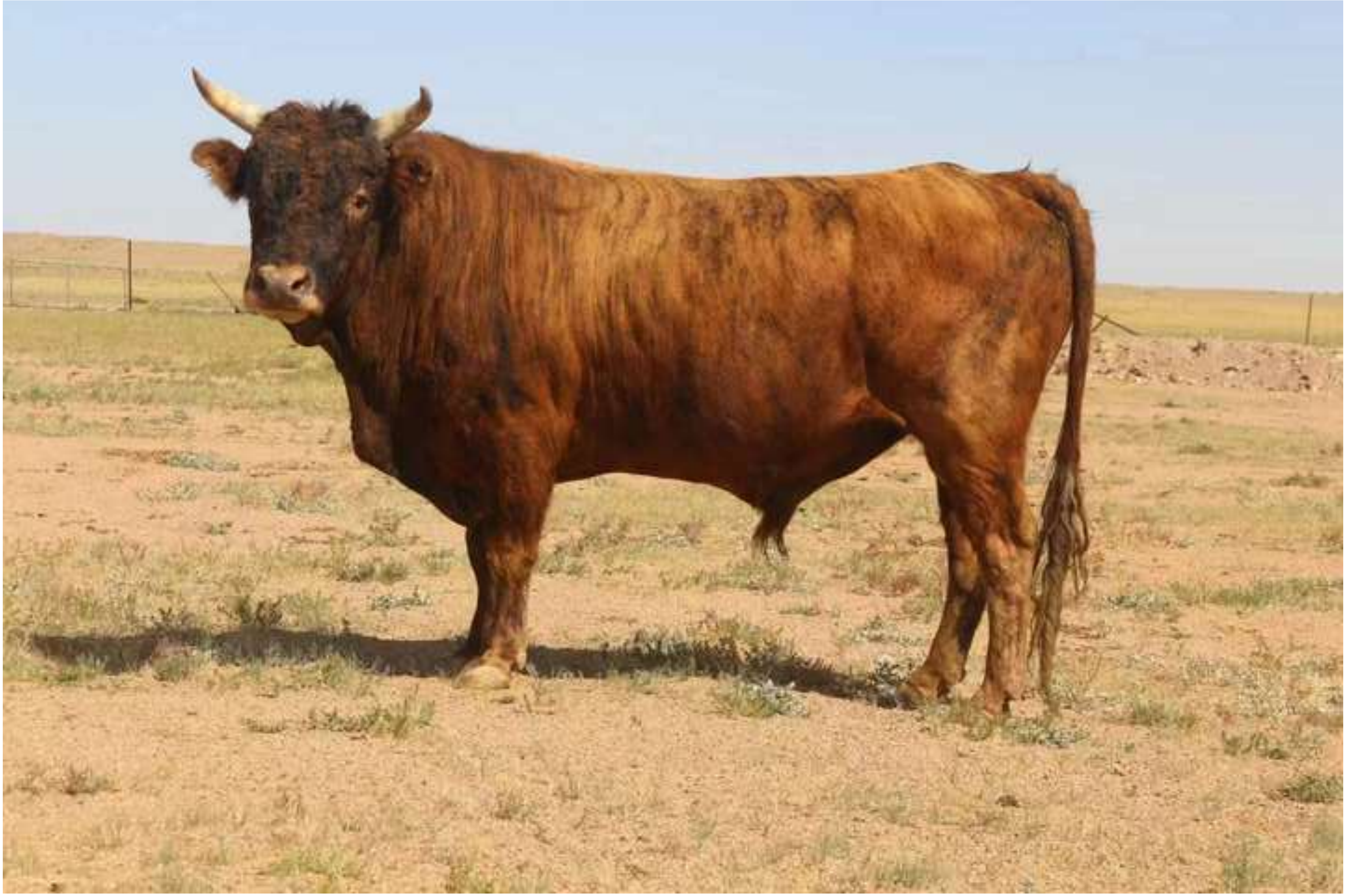

Click here to access/download;Figure;figure4-2.png

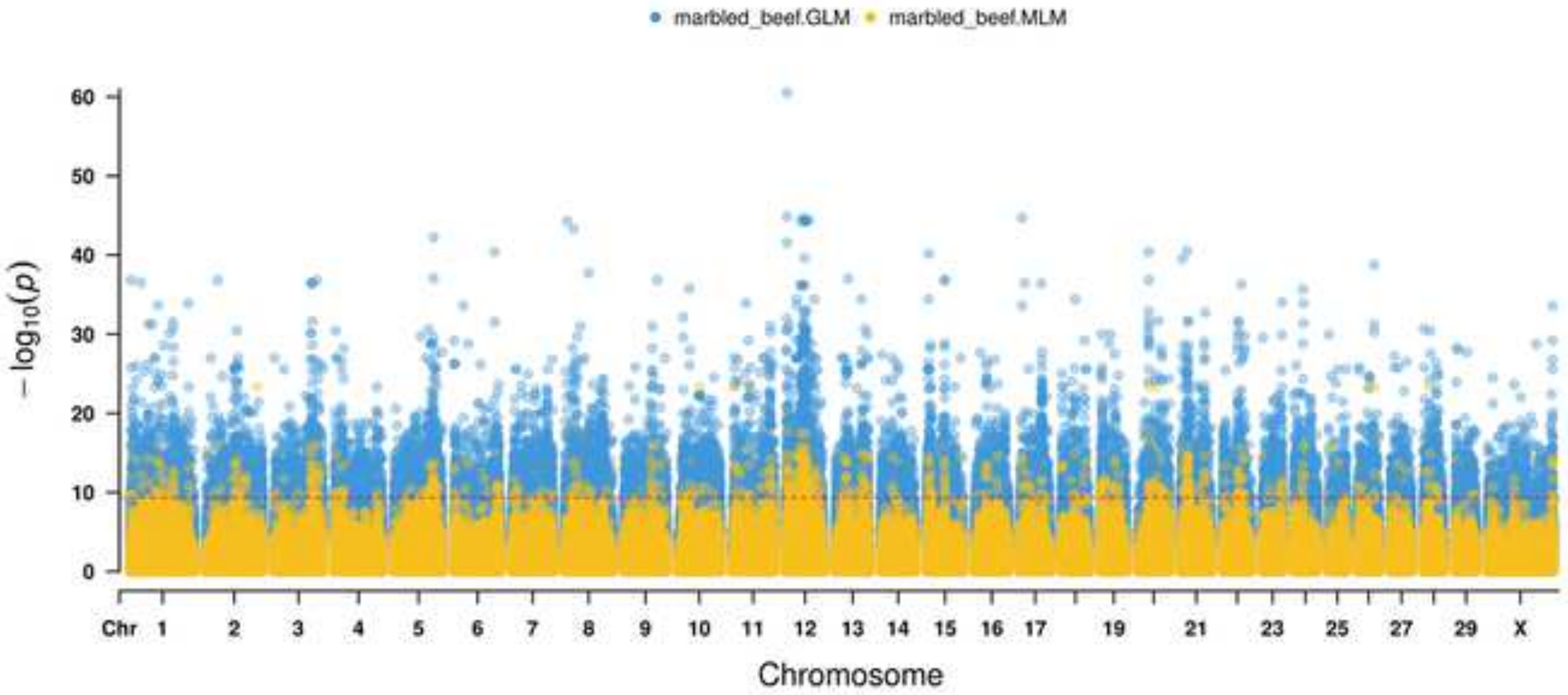

Click here to access/download;Figure;figure2.png

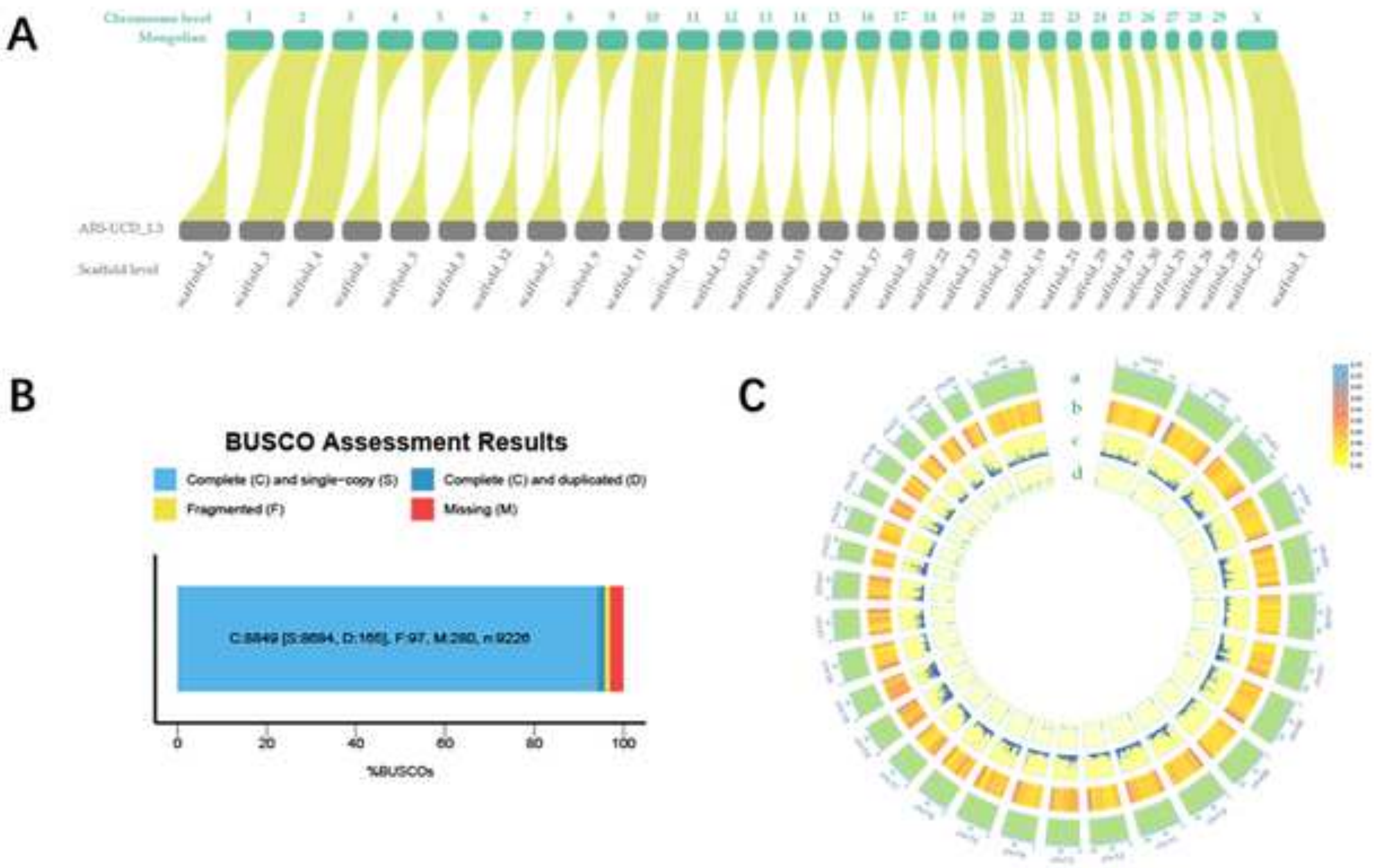

Click here to access/download
Supplementary Material
Table S1-information of raw data.xlsx

Click here to access/download
Supplementary Material
Table S2-assembly.xlsx

Click here to access/download
Supplementary Material
Figure S2 Ragtag_YvsOther.pdf

Click here to access/download
Supplementary Material
Figure S3.centromics_circos.pdf

Click here to access/download
Supplementary Material
Table S3-scaffold.xlsx

Click here to access/download
Supplementary Material
Table S4-QV-assessment.xlsx

Click here to access/download
Supplementary Material
Table S5-95_Individuals .xls

Click here to access/download
Supplementary Material
Table S6-information of cattle population.xlsx

Click here to access/download
Supplementary Material
Table S7-Mong_vs_Wagyu.xlsx

Click here to access/download
Supplementary Material
Table S8-Wagyu_vs_other.xlsx

Click here to access/download
Supplementary Material
Table S10-Wagyu_vs_other-gwas.xlsx

Click here to access/download
Supplementary Material
Figure-S1.Mongolian_spectra-cn plots.fl.pdf
