# Supplementary material for: Near telomere-to-telomere genome assembly of Mongolian cattle: implications for population genetic variation and beef quality
Source: Gigascience. 2024 Dec 18;13:giae099. doi: 10.1093/gigascience/giae099 (PMC11653892; doi:10.1093/gigascience/giae099)
Supplement: giae099_Response_to_Reviewer_Comments_Original_Submission [file giae099_response_to_reviewer_comments_original_submission.pdf]

Author's Response To Reviewer Comments

Dear Editor and Reviewers:

Thank you for the opportunity to revise our manuscript, "Near Telomere-to-Telomere Genome Assembly of Mongolian Cattle: Implications for Population Genetic Variation and Beef Quality" (GIGA-D-24-00289). We appreciate the reviewers' constructive feedback and valuable suggestions, and we are committed to addressing all the points raised to improve the quality of our work.
We will carefully address each of the reviewers' comments in a point-by-point manner. For any revisions that we do not agree with, we will provide detailed explanations and justifications. Additionally, we will ensure that our revised manuscript complies with the journal's style guidelines.
Thank you again for considering our manuscript for publication in GigaScience.

Best wishes,
He Meng

Reviewer #1: Su et al. present a high-quality Mongolian cattle genome with long reads and Hi-C data. The genome supersedes the current reference genome ARS-UCD1.3 and other published genome at NCBI, with higher continuity and completeness.
Response：Thank you for your positive feedback on our work. We are pleased that our high-quality Mongolian cattle genome, assembled with long reads and Hi-C data, surpasses the current reference genome ARS-UCD1.3 and other published genomes at NCBI in terms of continuity and completeness. We hope that these data will provide strong support for future genetic research and breeding efforts.
1. However, according to the description in the manuscript, only three chromosomes have telomere at both ends. No QV and coverage assessment have been performed. In addition, the process of Y chromosome assembling may be problematic since Y chromosome between species and even individuals could be highly variable in structure.
Response：Thank you for your valuable suggestions. We have added QV assessment to the manuscript and included the relevant figures in the supplementary materials in line 126-128 . As you pointed out, the assembly of the Y chromosome is not fully refined, we have reassembled the Y chromosome to improve the accuracy and revised the description of the Y chromosome assembly in the manuscript accordingly. We have compared it with those from other cattle breeds, showing high consistency and homology in line 129-131.
2. Besides, in Table S3 I notice that about 100 Mb sequence remained unanchored. Where should they belong? Please make some additional analysis.
Response：Thank you for pointing this out. We have analyzed the 100 Mb of unanchored sequence, and most of it consists of telomeric fragments. We did not include this information in the manuscript, as it is not the primary focus of our study and is a common phenomenon observed in genome assemblies. We appreciate your understanding on this matter.
3. In addition, the centromere presentation is largely missing in the manuscript. In the absence of throughout QC of the genome, I don't believe CentroMiner is a good tool to predict centromere candidates. The tool selects the tandem repeat monomers likely to be centromeric repeats on each chromosome based on period and copy number.
Response：Thank you for your valuable suggestion. We have added a description of the centromeres in the revised manuscript. Additionally, we utilized Centromics to enhance our predictions of the centromeres, and our results are consistent with those obtained using CentroMiner. (line 136-138)
4. Therefore if the centromere are collapsed in the genome, which usually happen during genome assembling, the use of CentroMiner would result in false positive predictions. Have the authors examined the monomer sequence?
Response: Thank you for your valuable suggestion. We have examined the centromere monomer sequences and performed the analysis accordingly. The result files have been uploaded for further review.(line136-138)
5. In the analysis of the Wagyu beef marbling trait, to show the advantage of the new genome, I would suggest generating a variation dataset with the current reference ARS-UCD1.3 as well with the same set of parameters and compare the dataset with the variation dataset of the Mongolian reference genome. I

doubt that the genes mentioned in the manuscripts can also be identified with ARS-UCD1.3. How many of them were newly reported in this study?
Response: Thank you for your valuable suggestion. While generating a variation dataset using the ARS-UCD1.3 reference could certainly provide useful comparative insights, our study is primarily focused on highlighting the distinctiveness and relevance of the Mongolian reference genome in relation to the Wagyu beef marbling trait. Taking into account both the scope of our research and computational considerations, we believe our current approach is the most suitable for the objectives of the study. We hope for your kind understanding.

Minor Review
1. Line132-138: liftoff only provide gene annotation transfer to the new genome, but would there be any new genes in the Mongolian genome?
Response: Thank you for your insightful suggestion. You are absolutely right that identifying novel genes in the Mongolian genome could provide additional valuable insights. However, given the completeness of current cattle genome annotations, we chose to focus on ensuring accurate gene annotation through Liftoff in this study. We believe this approach aligns best with the intended scope of our research. We appreciate your understanding.
2. Line 183: present the full name of GLM and MLM
Response: Thank you for your feedback. I have added the full names of GLM and MLM in the manuscript for clarity.
3. Line285-286, "Our study not only presents the first gapless bovine genome". There are at least 8 gaps in the genome and many scaffolds remained unanchored. Please revise.
Response: We have revised the statement to clarify that our study presents an improved bovine genome assembly characterized by fewer gaps compared to previous assemblies. We appreciate your suggestion in helping us enhance the clarity of our work.

Reviewer #2: In this manuscript, the authors provide a near telomere-to-telomere genome assembly of Mongolian cattle and a population genetic variation analysis. Most notably, the authors successfully assembled the Y chromosome and identified variant loci which may associate with the formation of marbling patterns in beef. Basically, this work is well described and the methods appropriate to the aims of the study. The description of dataset and workflows are clear and solid. However, the manuscript has some deficiencies that authors need to amend and address. Below are just the major and minor comments for this manuscript.
Due to the article type is data note, and there is no requirement of biological analyses for this manuscript. My recommendation on this manuscript is minor revision.
Response:
Thank you for your positive feedback and constructive comments regarding our manuscript. We are pleased to hear that you found our work on the near telomere-to-telomere genome assembly of Mongolian cattle and the population genetic variation analysis to be well described and methodologically appropriate. We appreciate your recognition of the clarity and solidity of our dataset and workflow descriptions. We will carefully consider your suggestions and address the deficiencies you have pointed out in our revision.
1. The Y chromosome is one of the most critical highlights of this manuscript, so the structure of Y chromosome is very important. Almost all the published papers which successful assembled the Y chromosome or just partial Y chromosome, all these papers expounded the structure Y chromosome. So, maybe the author can add this content, or at least compared the structure of Y chromosome between Mongolian cattle and other mammals.
Response: Thank you for your valuable suggestion regarding the importance of the Y chromosome structure in our manuscript. We have made revisions to enhance the discussion of the Y chromosome, including a comparative analysis of its structure between Mongolian cattle and the Y chromosomes of other bovine breeds (CM054900.2, CM037826.1, NC_082638.1, CP128563.1). This analysis aims to provide a clearer understanding of the Y chromosome's unique features (line129-132). We appreciate your insightful input and believe it has strengthened our manuscript.
2. Generally speaking, this manuscript didn't discover enough detail information or bright points. For example, the population genetic part was mostly enrichment analysis; it's a little rough and simple. We want to know the exactly gene and the specific mutation sites, which do associated with the formation of marbling patterns in beef.
Response：While we acknowledge that the population genetic analysis primarily focused on enrichment, we

also recognize the need for a deeper exploration. Therefore, we will provide detailed information on the specific genes and mutation sites associated with the formation of marbling patterns in beef , which can be found in Table S9. By including these elements, we aim to strengthen the overall analysis and contribute valuable insights to the field.

Minor point：

1. The BUSCO of the telomere-to-telomere genome is 95.9%, which is good for chromosome-level genome, but not good enough for the telomere-to-telomere genome. The authors should explain this. Incidentally, the database of BUSCO that the author used for assessment should be showed in manuscript.
Response: Thank you for your feedback. The BUSCO score of 95.9% reflects the difficulty in assembling repetitive regions and complex structures, even in near telomere-to-telomere assemblies. We have now included the BUSCO dataset (mammalia_odb10) in the manuscript for clarity.
2. The genome quality assessment is quite important for T2T assembly, only BUSCO assessment is definitely not enough. Maybe the synteny alignment between Mongolian cattle and other species (not only Hereford cattle), the structure of MHC, or sex chromosomes structure is needed.
Response: Thank you for your valuable suggestions. We have now included genome quality assessments using QV analysis assessment to provide a more comprehensive evaluation of the assembly(line 126-128).
3. The description of population variant analysis (Line 153-156) is quite confused. How the non-reference sequences were obtained? How did these non-reference sequences mapped back to the cattle reference genome?
Response: Thank you for your valuable suggestion. We have clarified the description of the population variant analysis in lines 153-158. The analysis was performed using the PopIns2 software, and a detailed explanation of the methodology can now be found in lines 258-269, outlining how non-reference sequences were obtained and mapped back to the cattle reference genome.
4. Line 153: It's population, not population.
Response: We have carefully reviewed the spelling of 'population' and confirmed that it is correct in the manuscript. Please let us know if there is another aspect you were referring to.
5. The thousands separators are always missing.
Resopnse: Thank you for your feedback. We have now added the thousands separators where they were previously missing.
