# Supplementary material for: Near telomere-to-telomere genome assembly of Mongolian cattle: implications for population genetic variation and beef quality
Source: Gigascience. 2024 Dec 18;13:giae099. doi: 10.1093/gigascience/giae099 (PMC11653892; doi:10.1093/gigascience/giae099)
Supplement: giae099_Response_to_Reviewer_Comments_Revision_1 [file giae099_response_to_reviewer_comments_revision_1.pdf]

Author's Response To Reviewer Comments

Dear Editor and Reviewers:

We are grateful for your suggestive comments. We have revised the manuscript carefully and made some corrections accordingly. Meanwhile the main corrections in the manuscript and the responses to the reviewer’s comments are attached.

Shanghai Jiao Tong University
He Meng

The authors have carefully revised the manuscript and addressed most issues. However, I'm still concerned about the Y chromosome assembling and further analysis. The authors mentioned that "For assembling the Y chromosome, the existing cattle genome Y chromosome sequence from NCBI (CP128563.1) served as the reference scaffold, and the assembly was further refined using RagTag". I believed that such a method may introduce reference bias in genome assembling therefore deeply doubt the current finding of "high c onsistency and homology". I would suggest de novo assembling the Y chromosome, because the Y chromosome structures are found largely diverse across different species and even across different individuals [10.1038/s41586-024-07473-2].
Response: Thank you for your valuable suggestions and for your interest in our research. We completely agree with your viewpoint that de novo assembly of the Y chromosome is preferable. However, due to our sequencing depth and the complexity of the Y chromosome, we opted for an alternative approach, which has yielded satisfactory results.
The reference scaffold we used is derived from a T2T-level Y chromosome (CP128563.1), which is considered a reliable benchmark. Additionally, we performed synteny analysis with Y chromosomes from other cattle breeds (see Figure S2), which showed that our assembled Y chromosome exhibits general consistency and homology with other breeds. Therefore, while our Y chromosome assembly is somewhat shorter and may not fully reflect the diversity across different breeds, we believe it is accurate and can serve as a reference.
In the future, we will take the sequencing depth into account and aim to assemble the Y chromosome de novo whenever possible. Thank you again for your insightful suggestions and guidance; we will carefully consider your recommendations.
Minor comments
1. Since the authors said that among the 100 Mb unanchored sequences, "most of it consists of telomeric fragments", please add such information in Table S3.
Response: Thank you for your suggestion. We have added the information regarding the composition of the 100 Mb unanchored sequences, specifically that "most of it consists of telomeric fragments," to Table S3. We appreciate your input, which has helped improve the clarity of our manuscript.
2. Please add figure legend to Fig. S3. What did the red and purple lines indicate? And from what result/figure/table can the reader believed that the Centromics result "are consistent with those obtained using CentroMiner"
Response: Thank you for your valuable feedback. We have now added a figure legend to Fig. S3. Additionally, the results from Centromics and CentroMiner have been included in Table S3 to demonstrate their consistency. We hope this addresses your concern and improves the clarity of the data presented.
3. To better promote the wider usage of the Mongolian genome, I would suggest author to provide a liftover chain file for the coordinate conversion between the new genome and ARS-UCD1.3.
Response: Thank you for your suggestio. We have now included the chain file as Supplementary Material File S1. Additionally, we have added a note regarding this in the manuscript at line 145 to guide readers on its availability and use.
