# Supplementary material for: Near telomere-to-telomere genome assembly of Mongolian cattle: implications for population genetic variation and beef quality
Source: Gigascience. 2024 Dec 18;13:giae099. doi: 10.1093/gigascience/giae099 (PMC11653892; doi:10.1093/gigascience/giae099)
Supplement: giae099_Reviewer_1_Report_Original_Submission — Yang Zhou -- 8/15/2024 [file giae099_reviewer_1_report_original_submission.pdf]

Reviewer Report

Title: Near Telomere-to-Telomere Genome Assembly of Mongolian Cattle: Implications for Population Genetic Variation and Beef quality

Version: Original Submission **Date:** 8/15/2024

Reviewer name: Yang Zhou

Reviewer Comments to Author:

Su et al. present a high-quality Mongolian cattle genome with long reads and Hi-C data. The genome supersedes the current reference genome ARS-UCD1.3 and other published genome at NCBI, with higher continuity and completeness.However, according to the description in the manuscript, only three chromosomes have telomere at both ends. No QV and coverage assessment have been performed. In addition, the process of Y chromosome assembling may be problematic since Y chromosome between species and even individuals could be highly variable in structure. Besides, in Table S3 I notice that about 100 Mb sequence remained unanchored. Where should they belong? Please make some additional analysis. In addition, the centromere presentation is largely missing in the manuscript. In the absence of throughout QC of the genome, I don't believe CentroMiner is a good tool to predict centromere candidates. The tool selects the tandem repeat monomers likely to be centromeric repeats on each chromosome based on period and copy number. Therefore if the centromere are collapsed in the genome, which usually happen during genome assembling, the use of CentroMiner would result in false positive predictions. Have the authors examined the monomer sequence?In the analysis of the Wagyu beef marbling trait, to show the advantage of the new genome, I would suggest generating a variation dataset with the current reference ARS-UCD1.3 as well with the same set of parameters and compare the dataset with the variation dataset of the Mongolian reference genome. I doubt that the genes mentioned in the manuscripts can also be identified with ARS-UCD1.3. How many of them were newly reported in this study?Minor comments:Line132-138: liftoff only provide gene annotation transfer to the new genome, but would there be any new genes in the Mongolian genome?Line 183: present the full name of GLM and MLM.Line285-286, "Our study not only presents the first gapless bovine genome". There are at least 8 gaps in the genome and many scaffolds remained unanchored. Please revise.

Level of Interest

Please indicate how interesting you found the manuscript: Choose an item.

Quality of Written English

Please indicate the quality of language in the manuscript: Choose an item.

Declaration of Competing Interests

Please complete a declaration of competing interests, considering the following questions:

- Have you in the past five years received reimbursements, fees, funding, or salary from an organisation that may in any way gain or lose financially from the publication of this manuscript, either now or in the future?
- Do you hold any stocks or shares in an organisation that may in any way gain or lose financially from the publication of this manuscript, either now or in the future?
- Do you hold or are you currently applying for any patents relating to the content of the manuscript?
- Have you received reimbursements, fees, funding, or salary from an organization that holds or has applied for patents relating to the content of the manuscript?
- Do you have any other financial competing interests?
- Do you have any non-financial competing interests in relation to this paper?

If you can answer no to all of the above, write 'I declare that I have no competing interests' below. If your reply is yes to any, please give details below.

I declare that I have no competing interests

I agree to the open peer review policy of the journal. I understand that my name will be included on my report to the authors and, if the manuscript is accepted for publication, my named report including any attachments I upload will be posted on the website along with the authors' responses. I agree for my report to be made available under an Open Access Creative Commons CC-BY license (http://creativecommons.org/licenses/by/4.0/). I understand that any comments which I do not wish to be included in my named report can be included as confidential comments to the editors, which will not be published.

Choose an item.

To further support our reviewers, we have joined with Publons, where you can gain additional credit to further highlight your hard work (see: https://publons.com/journal/530/gigascience). On publication of this paper, your review will be automatically added to Publons, you can then choose whether or not to claim your Publons credit. I understand this statement.

Yes Choose an item.
