# Supplementary material for: Near telomere-to-telomere genome assembly of Mongolian cattle: implications for population genetic variation and beef quality
Source: Gigascience. 2024 Dec 18;13:giae099. doi: 10.1093/gigascience/giae099 (PMC11653892; doi:10.1093/gigascience/giae099)
Supplement: giae099_Reviewer_1_Report_Revision_1 — Yang Zhou -- 9/30/2024 [file giae099_reviewer_1_report_revision_1.pdf]

Reviewer Report

Title: Near Telomere-to-Telomere Genome Assembly of Mongolian Cattle: Implications for Population Genetic Variation and Beef quality

Version: Revision 1 **Date:** 9/30/2024

Reviewer name: Yang Zhou

Reviewer Comments to Author:

The authors have carefully revised the manuscript and addressed most issues. However, I'm still concerned about the Y chromosome assembling and further analysis. The authors mentioned that "For assembling the Y chromosome, the existing cattle genome Y chromosome sequence from NCBI (CP128563.1) served as the reference scaffold, and the assembly was further refined using RagTag". I believed that such a method may introduce reference bias in genome assembling therefore deeply doubt the current finding of "high consistency and homology". I would suggest de novo assembling the Y chromosome, because the Y chromosome structures are found largely diverse across different species and even across different individuals [10.1038/s41586-024-07473-2].Minor comments1. Since the authors said that among the 100 Mb unanchored sequences, "most of it consists of telomeric fragments", please add such information in Table S3.2. Please add figure legend to Fig. S3. What did the red and purple lines indicate? And from what result/figure/table can the reader believed that the Centromics result "are consistent with those obtained using CentroMiner"3. To better promote the wider usage of the Mongolian genome, I would suggest author to provide a liftover chain file for the coordinate conversion between the new genome and ARS-UCD1.3.

Level of Interest

Please indicate how interesting you found the manuscript: Choose an item.

Quality of Written English

Please indicate the quality of language in the manuscript: Choose an item.

Declaration of Competing Interests

Please complete a declaration of competing interests, considering the following questions:

- Have you in the past five years received reimbursements, fees, funding, or salary from an organisation that may in any way gain or lose financially from the publication of this manuscript, either now or in the future?
- Do you hold any stocks or shares in an organisation that may in any way gain or lose financially from the publication of this manuscript, either now or in the future?

- Do you hold or are you currently applying for any patents relating to the content of the manuscript?
- Have you received reimbursements, fees, funding, or salary from an organization that holds or has applied for patents relating to the content of the manuscript?
- Do you have any other financial competing interests?
- Do you have any non-financial competing interests in relation to this paper?

If you can answer no to all of the above, write 'I declare that I have no competing interests' below. If your reply is yes to any, please give details below.

I declare that I have no competing interests

I agree to the open peer review policy of the journal. I understand that my name will be included on my report to the authors and, if the manuscript is accepted for publication, my named report including any attachments I upload will be posted on the website along with the authors' responses. I agree for my report to be made available under an Open Access Creative Commons CC-BY license (http://creativecommons.org/licenses/by/4.0/). I understand that any comments which I do not wish to be included in my named report can be included as confidential comments to the editors, which will not be published.

Choose an item.

To further support our reviewers, we have joined with Publons, where you can gain additional credit to further highlight your hard work (see: https://publons.com/journal/530/gigascience). On publication of this paper, your review will be automatically added to Publons, you can then choose whether or not to claim your Publons credit. I understand this statement.

Yes Choose an item.
