# Supplementary material for: Near telomere-to-telomere genome assembly of Mongolian cattle: implications for population genetic variation and beef quality
Source: Gigascience. 2024 Dec 18;13:giae099. doi: 10.1093/gigascience/giae099 (PMC11653892; doi:10.1093/gigascience/giae099)
Supplement: giae099_Reviewer_2_Report_Original_Submission — Yandong Ren, Ph.d -- 8/15/2024 [file giae099_reviewer_2_report_original_submission.pdf]

Reviewer Report

Title: Near Telomere-to-Telomere Genome Assembly of Mongolian Cattle: Implications for Population Genetic Variation and Beef quality

Version: Original Submission **Date:** 8/15/2024

Reviewer name: Yandong Ren, Ph.d

Reviewer Comments to Author:

In this manuscript, the authors provide a near telomere-to-telomere genome assembly of Mongolian cattle and a population genetic variation analysis. Most notably, the authors successfully assembled the Y chromosome and identified variant loci which may associate with the formation of marbling patterns in beef. Basically, this work is well described and the methods appropriate to the aims of the study. The description of dataset and workflows are clear and solid. However, the manuscript has some deficiencies that authors need to amend and address. Below are just the major and minor comments for this manuscript.Due to the article type is data note, and there is no requirement of biological analyses for this manuscript. My recommendation on this manuscript is minor revision.Major points:1. The Y chromosome is one of the most critical highlights of this manuscript, so the structure of Y chromosome is very important. Almost all the published papers which successful assembled the Y chromosome or just partial Y chromosome, all these papers expounded the structure Y chromosome. So, maybe the author can add this content, or at least compared the structure of Y chromosome between Mongolian cattle and other mammals.2. Generally speaking, this manuscript didn't discover enough detail information or bright points. For example, the population genetic part was mostly enrichment analysis; it's a little rough and simple. We want to know the exactly gene and the specific mutation sites, which do associated with the formation of marbling patterns in beef.Minor points:1. The BUSCO of the telomere-to-telomere genome is 95.9%, which is good for chromosome-level genome, but not good enough for the telomere-to-telomere genome. The authors should explain this. Incidentally, the database of BUSCO that the author used for assessment should be showed in manuscript.2. The genome quality assessment is quite important for T2T assembly, only BUSCO assessment is definitely not enough. Maybe the synteny alignment between Mongolian cattle and other species (not only Hereford cattle), the structure of MHC, or sex chromosomes structure is needed.3. The description of population variant analysis (Line 153-156) is quite confused. How the non-reference sequences were obtained? How did these non-reference sequences mapped back to the cattle reference genome?4. Line 153: It's population, not population.5. The thousands separators are always missing.

Level of Interest

Please indicate how interesting you found the manuscript: Choose an item.

Quality of Written English

Please indicate the quality of language in the manuscript: Choose an item.

Declaration of Competing Interests

Please complete a declaration of competing interests, considering the following questions:

- Have you in the past five years received reimbursements, fees, funding, or salary from an organisation that may in any way gain or lose financially from the publication of this manuscript, either now or in the future?
- Do you hold any stocks or shares in an organisation that may in any way gain or lose financially from the publication of this manuscript, either now or in the future?
- Do you hold or are you currently applying for any patents relating to the content of the manuscript?
- Have you received reimbursements, fees, funding, or salary from an organization that holds or has applied for patents relating to the content of the manuscript?
- Do you have any other financial competing interests?
- Do you have any non-financial competing interests in relation to this paper?

If you can answer no to all of the above, write 'I declare that I have no competing interests' below. If your reply is yes to any, please give details below.

I declare that I have no competing interests.

I agree to the open peer review policy of the journal. I understand that my name will be included on my report to the authors and, if the manuscript is accepted for publication, my named report including any attachments I upload will be posted on the website along with the authors' responses. I agree for my report to be made available under an Open Access Creative Commons CC-BY license (http://creativecommons.org/licenses/by/4.0/). I understand that any comments which I do not wish to be included in my named report can be included as confidential comments to the editors, which will not be published.

Choose an item.

To further support our reviewers, we have joined with Publons, where you can gain additional credit to further highlight your hard work (see: https://publons.com/journal/530/gigascience). On publication of this paper, your review will be automatically added to Publons, you can then choose whether or not to claim your Publons credit. I understand this statement.

Yes Choose an item.
